# Evaluation of long-read 16S rRNA next-generation sequencing for identification of bacterial isolates in a clinical diagnostic laboratory

**DOI:** 10.1128/jcm.01670-24

**Published:** 2025-04-22

**Authors:** Victoria L. Campodónico, Jean Ruelle, Anna Fitzgerald, Yehudit Bergman, Brenda Osborne, Dimitrios Bourdas, Jennifer Lu, Karen C. Carroll, Patricia J. Simner

**Affiliations:** 1 Department of Pathology, Johns Hopkins University School of Medicine 1500, Baltimore, Maryland, USA; 2 SmartGene Services, EPFL Innovation Park, Lausanne, Switzerland; 3 Department of Medicine, Johns Hopkins University School of Medicine 1500, Baltimore, Maryland, USA; Universitat Münster, Münster, Germany

**Keywords:** 16S rRNA, Nanopore, Sanger sequencing, identification

## Abstract

Sanger sequencing of the first ~500 bp of the 16S rRNA gene is frequently used to identify bacterial pathogens that have ambiguous biochemical profiles or proteomic mass spectra. When diversity does not occur within that region, genus-level and/or species-level identification may not be possible, and a longer sequence or alternative target may be required to distinguish between genera/species. In this study, we evaluated a clinically relevant end-to-end solution for long-read (~1,500 nt) 16S rRNA next-generation sequencing by Oxford Nanopore Technologies (ONT) compared to a ~500 nt Sanger sequencing approach for the identification of 153 bacterial clinical isolates. Sequencing data were analyzed using the IDNS software from SmartGene and its proprietary 16S Centroid reference database (Centroid database) SmartGene software and the Centroid database. The agreement of the two platforms on species- and genus-level identification was determined, and discrepancies were resolved by whole-genome sequencing. ONT had a higher taxonomic resolution at the genus level (*P* < 0.01). When genus-level identification was achieved by both methods, concordance to the best matching genus was 100%. When species-level identification was achieved by both methods, concordance to the best matching species was 91%. The costs per test were ~$25.30 (when multiplexing 24 samples/run) and $74 for ONT and Sanger sequencing, respectively. The hands-on time spent performing sequencing was similar for both methods, but the turnaround time of ONT was significantly shorter than that of Sanger sequencing.

**IMPORTANCE:**

This study adds to existing literature by describing a validated end-to-end solution of 16S rRNA gene Oxford Nanopore sequencing for bacterial isolate identification, including sequencing run time evaluation, automated analysis (SmartGene 16S Identification App) and interpretation of results, that can be incorporated into clinical and public health laboratories with a simple and cost-effective workflow.

## INTRODUCTION

16S rRNA gene sequencing is used in clinical laboratories for routine identification of bacterial pathogens that have ambiguous biochemical profiles or cannot be identified by matrix-assisted laser desorption-ionization time of flight mass spectrometry (MALDI-TOF MS) (1). The 16S rRNA gene is present in all bacteria, and it is highly conserved and evolves slowly. These factors make it the most widely used molecular target for genus- or species-level bacterial identification in the clinical laboratory (2). 16S rRNA Sanger sequencing (16S SS), which mainly focuses on the first ~500 bp (encompasses variable regions 1–3) of the 16S rRNA gene, offers high base-calling accuracy but it is costly, laborious, and time consuming, with a 2- to 3-day turnaround time and low throughput even with a multi-capillary approach (1, 3). When diversity does not occur within that region of the 16S rRNA gene, genus-level and/or species-level identification may not be possible by 16S SS. In these cases, the Clinical and Laboratory Standards Institute (CLSI) MM18 2A guidelines indicate that a longer section of the 16S rRNA gene or an alternative target may be required to achieve species-level identification (4). Long-read Nanopore sequencing (Oxford Nanopore Technologies [ONT]) can provide sequencing of the full-length (~1,500 bp, encompassing all nine variable regions) 16S rRNA gene in real time, offering a higher taxonomic resolution. Early versions of Nanopore sequencing technology using the R.9.4.1 flow cells had a relatively high read error rate of 5%–15%, which could hinder its accuracy for species-level identification (1, 5). However, the newest R10 sequencing chemistry incorporates “duplex base calling” and improves homopolymer calls providing Q30 (>99% accuracy) single-molecule reads (6).

Numerous studies have undertaken comparisons between 16S rRNA gene Oxford Nanopore sequencing (16S ONT) and second-generation next-generation sequencing (NGS) platforms like Illumina sequencing, showing that both technologies enable reliable identification of bacterial genera but may lead to inaccuracies in species-level identification. However, Nanopore appeared to be the preferred method for 16S rRNA gene amplicon sequencing when aiming for species-level taxonomic resolution, detecting rare taxa, or obtaining a precise estimation of richness in mixed samples (7–10). Studies comparing amplicon-based 16S ONT and 16S SS for diagnosing infectious diseases in mixed clinical specimens, such as pleural and ascites fluid, bronchoalveolar lavage fluid, urine, and wound secretions, showed that 16S ONT had a higher sensitivity than Sanger sequencing (11). However, there is a limited body of research specifically addressing the comparative analysis between 16S ONT and 16S SS for the identification of cultured bacterial isolates that cannot be definitively identified by MALDI-TOF MS, particularly in the clinical setting (1, 5, 12).

In this study, we evaluated long-read 16S rRNA next-generation sequencing by ONT compared to a ~500 nt Sanger sequencing approach for bacterial isolate identification. The costs and hands-on time of both platforms were also compared (13).

## MATERIALS AND METHODS

### Bacterial isolates

One hundred fifty-three bacterial clinical isolates were collected from the Johns Hopkins Hospital Microbiology Laboratory in Baltimore, MD, USA. One hundred and two isolates were prospective clinical isolates that could not be identified by MALDI-TOF MS and underwent 16S SS as part of the standard of care because they were considered clinically significant and not contaminants. Additionally, 51 selected bacterial clinical isolates within genera/species for which a longer 16S rRNA gene sequence is needed to resolve identification based on the CLSI MM18 A2 guidelines and that were available in our laboratory were selected as challenge organisms (Table S1) (4). The challenge organisms underwent 16S SS as part of this study if they had been identified using other methods (e.g., MALDI-TOF MS or cell wall fatty acid analysis using gas-liquid chromatography) and had not been sequenced previously.

### DNA extraction

To extract DNA for 16S SS, bacteria were re-suspended in PrepMan Ultra Sample Preparation Reagent (Applied BioSystems [ABS], Waltham, MA, USA) to create a lightly turbid solution (<0.5 McFarland) and boiled for 10 min. After centrifugation at 18,400 × *g* for 8 min, the supernatant thus obtained was used for PCR amplification.

For 16S ONT, boil extraction using the PrepMan Ultra Sample Preparation Reagent interfered with ONT sequencing. Thus, DNA was extracted using the Quick-DNA Fungal/Bacterial Miniprep kit (Zymo, Irvine, CA, USA). DNA concentration was determined using the Qubit 4 fluorometer (Invitrogen, Waltham, MA, USA) with the Qubit 1× double-stranded DNA (dsDNA) HS assay kit (Invitrogen), and the purity of the DNA was assessed by measuring the ratio of absorbance at 260 and 280 nm with a NanoDrop spectrophotometer (Thermo Fisher Scientific, Waltham, MA). DNA samples with a 260:280 ratio of ~1.8 were considered of acceptable purity.

### 16S rRNA Sanger sequencing

For 16S SS, the first 500 bp (V1–V3 region) of the 16S rRNA gene was sequenced using the MicroSEQ 500 16S rDNA PCR kit (ABS) (14) and the Applied Biosystems 3500 genetic analyzer (Thermo Fisher Scientific). The resulting raw trace files were analyzed using the ABS Sequence Scanner software (v.1.0) (Thermo Fisher Scientific). The sequence of each sample was classified by performing a Basic Local Alignment Search Tool (BLAST) search against the IDNS 16S Centroid database (SmartGene AG, Switzerland).

### 16S rRNA ONT sequencing

For 16S ONT, library preparation was performed using the 16S Barcoding Kit 1-24 (SQK-16S024; Oxford Nanopore Technologies, Oxford, UK) following the manufacturer recommendations. Sequencing was performed using new (not washed or reused) FLO-MIN111 flow cells (v.R10.3). For a subset of 44 samples, library preparation was additionally performed using the 16S Barcoding Kit 24 V14 (SQK-16S114.24, Oxford Nanopore Technologies) following the manufacturer recommendations with the R10.4.1 flow cells. Sequencing was performed with the GridION sequencer on the MinKNOW (v.21.11.7) platform using high-accuracy base calling (Guppy v.5.1.13) and standard filters. Each sequencing run consisted of 22 bacterial unknowns, 1 positive and 1 negative control. The passed FASTQ files generated were uploaded to the SmartGene Advanced Sequencing platform (SmartGene AG).

### Bioinformatics analysis: SmartGene’s Identification App for 16S

16S ONT FASTQ files were loaded into the Identification App from SmartGene. This Identification App is intended to detect and identify the predominant organism present in a primary sample or to identify an isolated strain. SmartGene’s Identification App for 16S automates the following steps:

1. Files are converted, and reads are filtered for quality to eliminate reads shorter than 20 nucleotides or having a mean Phred quality score below 7.
2. A subset of reads (here 1,000) is randomly selected, and a BLAST search is performed against the SmartGene 16S Centroid reference database. SmartGene’s 16S Centroid database is a proprietary database (patent #EP02215578) of non-redundant, representative, bacterial 16S rRNA sequences covering 16,420 species across 3,312 genera as of August 2023 (v.r144u553). To create and update the 16S Centroid database, SmartGene periodically extracts 16S rDNA sequence data from public repositories using specific, proprietary profiles to filter out unreliable sequences and annotations. The extraction algorithms for 16S accommodate the natural diversity of the gene for each species across the bacterial kingdom. Supplementary algorithms are then used to examine all 16S rDNA sequences extracted and retained in order to identify the most representative sequences for valid species names, according to current taxonomy (bacterio.net). Each such sequence selected is dubbed the “Centroid” for its species group and is included within the 16S Centroid reference database. Data elements (such as species group size) derived from the Centroid database creation process are added to each Centroid record. All sequences included in the database are linked to their public repository accession number for full traceability. Validation of each Centroid and its annotation is performed using artificial intelligence-driven algorithms. Frequent updates incorporate recently described organisms, new strains, and updated taxonomy.
3. The most abundant genus as represented by these 1,000 reads is determined. When input DNA comes from a pure culture of an isolated organism, a single genus or a clearly dominant genus is expected; if no genus is found which accounts for >50% of these 1,000 reads, the pipeline stops.
4. Centroid sequences for all species belonging to that specific genus are then extracted.
5. The Centroid sequences are aligned to create a genetic profile which is specific for that genus and reflective of the intra-genus diversity of the extracted 16S sequences.
6. All reads present in the original FASTQ file and which pass the quality filters are aligned against the profile to generate a consensus sequence for that sample. When determining the consensus sequence for the study samples, an interpretation cutoff of 40% was applied at each nucleotide position to exclude artifact ambiguities or read errors from the Nanopore sequencer, which are assumed to be stochastic, and, thus, to avoid corruption of the consensus sequence. The minimum number of reads per position was here set to five.
7. The consensus sequence is compared to the 16S Centroid database using a BLAST search.
8. A results table is generated and displays the five most closely matching species found, the table being sorted by percent identity and number of mismatches, together with a species attribution confidence score which consolidates metrics for match consistency, equivalence, accuracy, and quality.

### Establishing a minimum number of reads needed for a stable and reliable consensus

Eighty-four records with profile sequence alignment files available containing at least 1,000 reads were randomly selected from the sample records. For each record, a consensus was created using all reads available, using as parameters a minimum depth of five reads and an interpretation cutoff of 40%. This consensus, derived from all reads, was assigned as the expected consensus. The file passes were then reduced into smaller subsets: the first subset containing the first 500 reads for the sample, the second containing 1,000 reads (500 of the first file plus 500 next reads), and so on, until all reads for a sample were included up to a maximum of 20,500 reads. For each subset, a frequency matrix and a consensus (with the same parameters) were generated. The Hamming distance (HD) between each consensus and the expected consensus was then computed. HD is zero when sequences are identical and increases with distance, in direct relation with the number of mismatches between the consensus of a subset file and the expected consensus. To establish the minimum number of reads required for a stable identification, the sum of all HDs was plotted against the number of reads (in increments of 500 reads), together with the number of identical consensus sequences for each of those 500-read increments. The points where the curves flatten indicate the stability of the consensus sequences (Fig. S4). Since the 84 records considered had different numbers of total reads, the plots were repeated to provide a comparison between the subsets of records having at least 10,000 reads and those having at least 15,000 reads.

### Determination of minimum sequencing run time required for the 16S ONT workflow

To determine the minimal sequencing run time required as part of the 16S ONT workflow, 138 sequence files having at least 500 reads were selected to determine the time to reach 500 reads and to compare the number of mismatches and the identification achieved by analyzing the consensus obtained with 500 reads vs. the one obtained with the total number of reads after 72 h of sequencing.

### Taxonomic classification and reporting of results

The level of identification was determined following CLSI MM18 2A guidelines for interpretation (4, 15). When not meeting the CLSI identity cutoffs for species- and genus-level identification, family-level identification was given to the top matched organism with ≥95% identity, and an organism was determined unable to be identified when the percent identity was below 95%. In addition, because the CLSI MM18 2A guidelines require a distance score greater than 0.4% to the next closest species in addition to ≥99.6% identity to achieve identification to the species level of aerobic actinomycetes, the interpretation of the sequencing results from those organisms was compared to results obtained using modified CLSI guidelines where species-level identification was awarded to the top matched species with ≥99.6% identity irrespective of the similarity score differences to the next closest species match (15).

Additionally, the top classified taxa obtained from 16S SS and 16S ONT data were compared, and the species-level concordance was calculated. When species-level identification was not obtained by either method, genus- or family-level identification was determined and compared. Matched species were excluded if the match length was <400 bp for 16S SS or <1,250 bp for 16S ONT.

### Whole-genome sequencing

For discrepant results between 16S SS and 16S ONT, whole-genome sequencing (WGS) was pursued. DNA was extracted using the Quick-DNA Fungal/Bacterial Miniprep kit. NGS libraries were prepared using Illumina DNA Prep. Library concentration was verified using a dsDNA fluorescent dye method, Qubit (v.3.0) Fluorometer (Thermo Fisher Scientific). DNA fragment size and library quality were confirmed on a 4200 TapeStation system (Agilent, CA, USA). Sequencing was performed on the Illumina NextSeq instrument using the NextSeq 1000/2000 P2 Reagents (300 cycles) (v.3) kit (Illumina, San Diego, CA, USA).

All isolates were sequenced to yield an average of 590,000 300 bp paired reads (150 bp per mate). The paired reads were then loaded into the PHoeNIx bioinformatics pipeline for short-read analysis and species identification. The PHoeNIx (https://github.com/CDCgov/phoenix) pipeline automates the following steps for Illumina paired-end reads: (i) PhiX174 reads and adapters are removed using BBDuK (https://github.com/BioInfoTools/BBMap); (ii) reads undergo filtering and trimming using fastp (https://github.com/OpenGene/fastp) based on FASTQ quality scores and the length of each read; (iii) the filtered and trimmed reads are then assembled using the SPAdes genome assembler (https://currentprotocols.onlinelibrary.wiley.com/doi/abs/10.1002/cpbi.102). Assembled contigs smaller than 500 bp are filtered and removed; (iv) contamination and species are identified using Kraken2 classification against a standard human and RefSeq bacterial/viral/archaeal database (16); (v) final bacterial identification of each isolate is provided by calculating the sequence distance from all complete RefSeq bacteria using Mash (17) and FastANI (18).

### Statistical analysis

The accuracy of bacterial identification by 16S SS and 16S ONT was compared using McNemar’s test, a test for paired proportions (19); by this method, *P* values of <0.05 indicate a statistically significant difference.

## RESULTS

### Number of reads and mismatches

The Nanopore platform generated an average of 20,029 reads (range 68–300,523) per sample. There was no correlation between the number of reads obtained per sample and the initial DNA concentration (Fig. S1). Among the bacterial groups examined, the aerobic actinomycetes and mycobacteria exhibited the most modest read counts, followed by the pigmented gram-negative rods (Fig. S2).

Sequencing of 17 isolates with 12–50 mismatches was repeated using the same DNA extracts to understand if sequencing would improve on repeat testing. While the number of reads obtained for each sample changed on the second sequencing run, the number of mismatches was almost identical for all of them, and the identifications did not change (Table S2). Additionally, five samples that produced only 68, 212, 576, 714, and 906 reads were repeated using the previously sequenced DNA extracts. Repeat sequencing resulted in 920, 4,000, 2,326, 13, and 455 reads, respectively. A consensus sequence was not obtained for the sample with only 13 reads, and it could not be re-analyzed. For three of the other samples, the interpretation (percent identity) and mismatches obtained did not change on repeat testing. The sample with 68 reads was identified as *Mycobacterium paragordonae* with 99.00% identity followed by *Mycobacterium gordonae* with 98.86% identity. However, after resequencing, 920 reads were obtained, and the identification changed to *M. gordonae* with 99.12% identity followed by *M. paragordonae* (99.00%) (Table S3). The total number of reads and mismatches of each sample by 16S SS and 16S ONT is shown in Table S1.

### Level of identification and concordance in bacterial speciation

**Fig 1:**
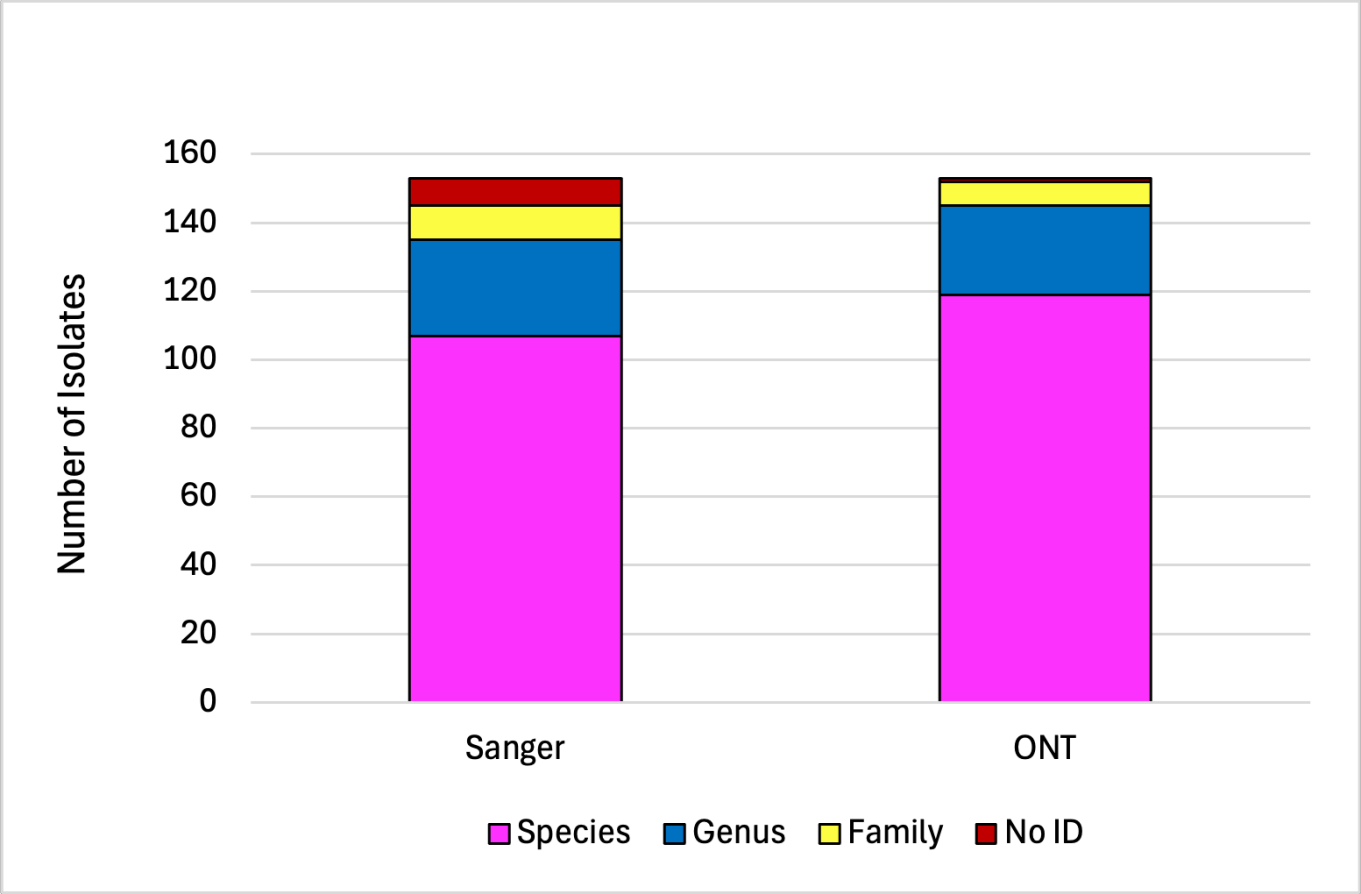
Level of identification obtained for all isolates sequenced by 16S SS and 16S ONT following CLSI MM18 2A guidelines for interpretation (*n* = 153).

Overall, applying the CLSI MM18 2A guidelines for interpretation, we found that 16S SS provided 67% (103 of 153) species-level, 21% (32 of 153) genus-level, and 7% (10 of 153) family-level identifications. 16S ONT significantly improved the identification from no ID or family to genus levels (*P* < 0.01) and from no ID to family level (*P* < 0.02) and provided 73% (111 of 153), 22% (34 of 153), and 5% (7 of 153) of species-, genus-, and family-level identifications, respectively. 16S SS was unable to identify eight (5%) isolates, while 16S ONT did not identify one (< 1%) (Table 1; Table S1; Fig. 1). For the prospective organisms, while 16S ONT significantly increased the identification of bacterial organisms from unable to ID or family- to genus-level identification (*P* < 0.02), it did not provide better identification from genus to species levels (*P* > 0.05, Table S4). Conversely, for the challenge organisms, 16S ONT improved the identification from genus to species levels (*P* < 0.03) but not from unable to ID or family to genus levels (*P* > 0.05), Table S5.

**TABLE 1:** Correlation between 16S SS and 16S ONT sequencing results for prospective and challenge isolates following CLSI MM18 2A guidelines for interpretation (*n* = 153)

		Identification by 16S Sanger sequencing			
		No ID (*n* = 8) (5%)	Family (*n* = 10) (7%)	Genus (*n* = 32) (21%)	Species (*n* = 103) (67%)
Identification by 16S ONT	No ID (*n* = 1) (<1%)	1 Anaerobic gram-positive cocci			
	Family (*n* = 7) (5%)	2 *Peptoniphilaceae* *Desulfovibrionaceae*	4 *Peptoniphilaceae Paracoccaceae* *Microbacteriaceae* *Nocardiaceae*	1 *Gulosibacter*	
	Genus (*n* = 34) (22%)	4 *Anaerococcus* *Butyricimonas* *Desulfovibrio* *Mixta*	6 *Myroides* *Pseudoxanthomonas* (×3) *Schaalia* *Nocardia*	16 *Corynebacterium* *Brevibacillus* *Massilia* *Roseomonas* *Porphyromonas* *Anaerococcus* *Mixta* *Mycobacterium* (×5) *Actinomadura* *Gordonia* *Nocardia* (×2)	8 *Moraxella canis* *Nocardia kruczakiae* *Dietzia maris* *Rhodococcus kroppenstedtii* (×2) *Streptomyces diastaticus* *Mycobacterium phocaicum*/*mucogenicum Mycobacterium lentiflavum*
	Species (*n* = 111) (73%)	1 *Brucella intermedia*		15 *Lysinibacillus boronitolerans* *Bosea robiniae* *Pseudoxanthomonas kaohsiungensis* *Cosenzaea myxofasciens* *Varibaculum cambriense* *Paenibacillus selenitireducens* *Aggregatibacter actinomycetemcomitans* *Brevibacterium paucivorans* *Klebsiella grimontii*^*a*^ *Cellulomonas pakistanensis* *Edwardsiella tarda* *Peptoniphilus asaccharolyticus* *Slackia exigua* *Mycobacterium arcueilense* *Mycobacterium gastri*	95

^*a*^ Species-level ID of *Klebsiella grimontii* would be reported as part of the *Klebseilla oxytoca* complex as 16S rRNA does not discern between members of this complex.

The concordance of the top species identified by each platform was 89% (51 of 57) and 92% (35 of 38) for the prospective and challenge organisms, respectively. The concordance for the top genus and family was 100% for both groups of organisms.

For the aerobic actinomycetes (*n* = 15), following the CLSI MM18 2A guidelines for interpretation resulted in 60% and 33% species-level identification for the 16S SS and 16S ONT, respectively, and there was 100% (four out of four) concordance on best matching species. Following the modified CLSI guidelines to ignore the distance to the next closest match, 16S SS and 16S ONT identified 80% (12/15) and 67% (10/15) of the organisms to the species level, respectively, with a 75% (9 of 12) concordance on the best matching species. The three organisms that were identified at the species level by 16S SS and at the genus level by 16S ONT when the CLSI MM18 2A guidelines were applied were identified as different species by 16S SS and 16S ONT when applying the modified CLSI guidelines (Table S6; Fig. S3).

Within the *Mycobacterium* spp. (*n* = 9), both methods provided the same level of identification, with five isolates identified at the genus level and four isolates identified at the species level by one method and at the genus level by the other one (Table S7).

Within the gram-positive isolates, 16S SS and 16S ONT identified 7 out of 12 (58%) and 9 out of 12 (75%) coryneform gram-positive rods at the species level, respectively, with 57% (four out of seven) concordance on the best matching species (Table S8). Gram-positive cocci (*n* = 10) were all identified at the species level by both methods, with 100% concordance for the best matching species. For endospore-forming aerobic gram-positive rods (*n* = 8), both methods provided species-level identification for five isolates, with 60% concordance for the best matching species, and genus-level identification for one. None of these differences were statistically significant according to McNemar’s test.

Within the gram-negative rods (*n* = 61), although 16S ONT provided species-level identification to six more isolates than 16S SS (51 vs. 45), this difference was not statistically significant. However, 16S ONT significantly improved the identification from no-ID/family level to genus level (*P* < 0.05), providing genus-level identification for six more isolates than 16S SS (Table S9).

Within the 38 anaerobic organisms tested, 16S SS and 16S ONT identified 25 (66%) and 28 (74%) organisms at the species level, 5 (13%) and 6 (16%) at the genus level, and 2 (5%) and 3 (8%) at the family level, and 6 (16%) and 1 (3%) were not identified, respectively. For the 25 isolates that were identified at the species level by both methods, there was a 96% correlation for the best matching species. Three isolates that were not identified by 16S SS and one that was identified at the family level were identified at the genus level by 16S ONT, and two isolates not identified by 16S SS were identified at the family level by 16S ONT (Table S10). However, these improvements in identification were not statistically significant.

### ONT sequencing run time and number of reads required to achieve a stable consensus and reliable identification

Eighty-four random samples were analyzed to determine how many reads were required to obtain a stable consensus. For 67 (80%) samples, the consensus achieved with 6,000 reads was the same as the consensus obtained with the total number of reads. Seventeen (20%) samples had a different consensus (maximum of 4 bp differences), but none of these differences changed the identification (Fig. S4). The sequencing time required for obtaining 6,000 reads varied significantly between the samples from 10 min to more than 1.5 days, and it negatively correlated with the number of reads present in the FASTQ file at the end of the 72 h of sequencing. To determine whether fewer reads could be used to obtain a reliable identification even if the consensus was not stable, the time to reach 500 reads for 138 samples was determined. It took a mean time of 2 h 16 min and 8 seconds to reach 500 reads per sample (minimum time: 2.07 min, maximum time: 58 h 50 min) in the configuration used during the study (batch of 24 samples). However, 91% (126 of 138) of the samples achieved 500 reads in less than 2 h. The remaining 12 samples required between 3 h 25 min and 58 h 50 min to achieve 500 reads. At the end of the 72 h set for sequencing, none of these 12 samples achieved 2,000 reads. These 12 samples included 1 non-lactose fermenting gram-negative rod, 5 aerobic actinomycetes, 2 anaerobic actinomycetes, 3 anaerobic gram-positive rods, and 1 gram-positive coccus (Table S10). For 97 samples (70%), the consensus obtained with 500 reads was the same as the one obtained with all the reads. Forty-one (30%) samples had a different consensus; 40 of them had between 1 and 5 bp differences, and that did not change the best matching species identified or the level of identification. For one sample with a 12 bp difference, the consensus obtained with 500 reads was not stable, and the best matching species identified changed, but the level of identification did not change and was at the genus level (*Butyricimonas* spp.).

### Whole-genome sequencing for bacterial isolates with discrepant species-level identification

Sixteen isolates that were identified at the species level by both methods but were assigned different top species by each method and one sample identified at the species level by 16S SS and at the genus level by 16S ONT were analyzed by WGS to identify the definite taxa (Table 2). Unfortunately, for 16 of the isolates, the best matching species assigned by one or the other 16S sequencing method were not in the WGS database used for WGS analysis, and therefore, even though a bacterial species was identified by WGS, we were not able to confidently resolve the discordant result. The remaining isolate identified as *Nocardia nova* by WGS was identified as *Nocardia kruczackiae* (100% identity) by 16S SS and as *Nocardia elegans* (99.59%) by 16S ONT. Both *N. kruczakiae* and *N. elegans* are species within the *N. nova* complex. Therefore, a definitive bacterial taxon could not be confirmed for any of these species after WGS discrepant analysis, and the final data comparison between 16S SS and 16S ONT was based on CLSI MM18 2A interpretation guidelines.

**TABLE 2:** Whole-genome sequencing results for bacterial isolates with discrepant species-level identification

16S ss	Identity (%)	Match length	Sequence length	16S ONT	Identity (%)	Match length	Sequence length	WGS	WGS comments
*Bacillus cereus* *Bacillus wiedmannii* *Bacillus paramycoides* *Bacillus thuringiensis* *Bacillus albus*	99.81	520	1,558 1,540 1,509 1,558 1,509	*Bacillus proteolyticus* *Bacillus (Weizmannia) acidiproducens* *Bacillus cereus* *Bacillus thuringiensis* *Bacillus luti*	99.93 99.93 99.87 99.87 99.87	1,513 1,452 1,514 1,514 1,514	1,513 1,452 1,558 1,558 1,558	*Bacillus cereus*	*B. proteolyticus* and *B. acidoproducens* are not in the database. There are no complete genomes for those species. *Bacillus luti* is in the database.
*Sphingomonas olei* *Sphingomonas faeni* *Sphingomonas aurantiaca*	99.78 98.48 98.06	446 460 464	1,435 1,483 1,452	*Sphingomonas mucosissima* *Sphingomonas olei* *Sphingomonas panaciterrae*	99.85 99.79 99.14	1,364 1,435 1,395	1,392 1,435 1,395	*Sphingomonas* sp.	None of the *Sphingomonas* spp. identified by 16S SS and 16S ONT are in the database.
*Dietzia maris* *Dietzia natronolimnacea*	99.60 98.60	501 500	1,483 1,511	*Dietzia kunjamensis^a^* *Dietzia maris*	99.93 99.86	1,439 1,480	1,439 1,483	*Dietzia kunjamensis*	
*Rhodococcus kroppenstedtii* *Rhodococcus corynebacteroides*	100 99.58	481 480	1,485 1,494	*Rhodococcus corynebacteroides^a^* *Rhodococcus kroppenstedtii* *Rhodococcus trifolii*	99.66 99.39 99.05	1,462 1,467 1,364	1,494 1,485 1,364	*Rhodococcus* sp.	Neither *R. kroppenstedtii* nor *R. corynebacteroides* is in the database.
*Rhodococcus kroppenstedtii* *Rhodococcus corynebacteroides*	99.79 99.37	475 475	1,485 1,494	*Rhodococcus corynebacteroides^a^* *Rhodococcus kroppenstedtii* *Rhodococcus trifolii*	99.67 99.39 99.05	1,494 1,467 1,364	1,494 1,485 1,364	*Rhodococcus* sp.	
*Stenotrophomonas bentonitica* *Stenotrophomonas rhizophila*	99.01 98.87	529 506	1,551 1,469	*Stenotrophomonas rhizophila* *Stenotrophomonas bentonitica*	99.67 99.52	1,509 1,469	1,551 1,469	*Stenotrophomonas rhizophila*	*S. bentonitica* is not in the database.
*Bosea vestrisii* *Bosea eneae* *Bosea thiooxidans*	99.35 98.92 98.92	463	1,452 1,452 1,475	*Bosea robiniae* *Bosea lupini*	99.09 99.08	1,429 1,412	1,439 1,414	*Bosea* sp.	None of the *Bosea* spp. identified by 16S SS and 16S ONT are in the database.
*Bosea vestrisii* *Bosea eneae* *Bosea thiooxidans*	99.36 98.94 98.94	472	1,452 1,452 1,475	*Bosea robiniae* *Bosea lupini*	99.09 99.08	1,431 1,412	1,439 1,414	*Bosea* sp.	
*Erwinia persicina* *Erwinia aphidicola* *Erwinia billingiae* *Erwinia rhapontici* *Erwinia rwandensis* *Erwinia tasmaniensis*	99.43 99.21 98.49 98.12 98.11 97.74	530 506 530 532 530 530	1,547 1,493 1,547 1,546 1,541 1,546	*Erwinia aphidicola* *Erwinia persicina*	99.73 99.47	1484 1522	1,493 1,547	*Erwinia persicina*	*Erwinia aphidicola* is not in the database.
*Lelliottia amnigena* *Lelliottia jeotgali* *Raoultella planticola* *Raoultella ornithinolytica* *Buttiauxella izardii*	100 99.59 98.78 98.78 98.58	492	1,498 1,536 1,541 1,541 1,498	*Lelliottia aquatilis* *Lelliottia nimipressuralis* *Lelliottia amnigena* *Buttiauxella izardii*	99.80 99.44 99.20 99.06	1,503 1,434 1,498 1,488	1,536 1,446 1,498 1,498	*Lelliottia* sp.	*Lelliottia amnigena*, *L. jeotgali*, and *L. nimipressuralis* are all in the database. *Lelliottia aquatilis* is not in the database.
*Nocardia kruczakiae* *Nocardia elegans* *Nocardia cerradoensis* *Nocardia mikamii* *Nocardia veterana* *Nocardia africana*	100 99.58 99.58 99.58 99.58 99.36	471	1,439 1,481 1,462 1,466 1,494	*Nocardia elegans^a^* *Nocardia cerradoensis* *Nocardia kruczakiae* *Nocardia mikamii* *Nocardia africana*	99.59 99.45 99.38 99.25 99.18	1,468 1,459 1,441 1,459 1,462	1,481 1,462 1,439 1,466 1,492	*Nocardia nova*	None of the *Nocardia* spp. identified by 16S SS and 16S ONT are in the database.
*Ochrobactrum ciceri* ^*b*^ *Ochrobactrum intermedium* ^*b*^ *Brucella inopinata* ^*b*^ *Ochrobactrum haematophilum* ^*b*^ *Ochrobactrum teleogrylli* ^*b*^ *Brucella microti* ^*b*^ *Brucella ceti* ^*b*^ *Brucella vulpis* ^*b*^ *Brucella melitensis* ^*b*^ *Brucella pinnipedialis* ^*b*^ *Brucella endophytica* ^*b*^ *Ochrobactrum daejeonense* ^*b*^	100 100 99.65 99.30 99.30 99.30 99.30 99.30 99.30 99.30 99.30 99.30	286*^b^* 285 286 286 286 286 286 286 286 286 285 285	1,400 1,479 1,545 1,475 853 1,489 1,489 1,489 1,489 1,489 1,417 1,431	*Ochrobactrum ciceri* ^*b*^ *Brucella intermedia*	100 99.03	1,243 1,449	1,400 1,479	*Brucella intermedia*	None of the *Ochrobactrum* spp. identified by 16S SS and 16S ONT are in the database. *Brucella ceti*, *B. microti*, *B. melitensis*, and *B. pinnipedialis* are in the database.
*Mycobacterium septicum* *Mycobacterium peregrinum* *Mycobacterium lutetiense* *Mycobacterium fortuitum* *Mycobacterium alvei*	99.80^*a*^ 99.60 99.60 99.20 99.20	503	1,487 1,471 1,482 1,486 1,524	*Mycobacterium arcueilense* *Mycobacterium peregrinum* *Mycobacterium septicum* *Mycobacterium montmartrense* *Mycobacterium lutetiense*	100.0 99.86 99.66 99.52 99.46	1,462 1,480 1,480 1,460 1,470	1,462 1,482 1,487 1,460 1,471	*Mycobacterium fortuitum*	None of the *Mycobacterium* spp. identified by 16S SS and 16S ONT are in the database.
*Psychrobacillus psychrodurans* *Psychrobacillus soli* *Psychrobacillus glaciei* *Psychrobacillus lasiicapitis*	99.06 99.04 98.88 97.95	531 523 536 536	1,520 1,484 1,520 1,518	*Psychrobacillus soli* *Psychrobacillus glaciei* *Psychrobacillus psychrodurans*	99.73 99.47 99.14	1,484 1,510 1,516	1,484 1,520 1,520	*Psychrobacillus glacei*	*Psychrobacillus glaciei* is the only named *Psychrobacillus* sp. in the database.
*Leifsonia shinshuensis* *Leifsonia soli* *Leifsonia poae* *Leifsonia lichenia*	99.79 99.59 98.77 98.56	487	1,528 1,398 1,487 1,504	*Leifsonia soli* *Leifsonia lichenia* *Leifsonia shinshuensis*	99.86 99.26 99.14	1,398 1,486 1,520	1,398 1,504 1,528	*Leifsonia shinshuensis*	*Leifsonia soli*, *L. poae*, and *L. lichenia* are not in the database.
*Pseudoglutamicibacter albus* *Pseudoglutamicibacter cumminsii*	99.37 99.15	475 471	1,501 1,483	*Pseudoglutamicibacter cumminsii* *Pseudoglutamicibacter albus*	99.73 99.46	1,472 1482	1,483 1,501	*Pseudoglutamicibacter albus*	*Pseudoglutamicibacter cumminsii* is not in the database.
*Moraxella canis*	99.80	509	1,507	*Moraxella porci* *Moraxella canis*	98.83 98.80	1,364 1,495	1,367 1,507	*Moraxella catarrhalis*	*Moraxella porci* and *Moraxella canis* are not in the database.

^*a*^ Genus-level identification.

^*b*^ Matched species were excluded because match length was <400 bp for 16S SS and <1,250 bp for 16S ONT.

### Whole-genome sequencing for bacterial isolates difficult to identify

Five bacterial isolates with <97% identity and one aerobic actinomycete with <99% identity by both sequencing methods were whole genome sequenced to identify definite taxa (Table 3). One isolate was identified as *Desulfovibrio fairfieldensis* (99.28%) by WGS. This taxonomic name has been effectively published, but not validly published, under the rules of the International Code of Nomenclature of Prokaryotes (Bacteriological Code) (https://www.ncbi.nlm.nih.gov/Taxonomy/Browser/wwwtax.cgi). Therefore, there is no Centroid sequence for this species in the Centroid database, and the closest match by 16S SS and 16S ONT was *Desulfovibrio desulfuricans* with 94.85% and 96.83% identities, respectively. An isolate for which the closest match was *Stenotrophomonas tumulicola* (96.81%) by 16S SS showed two different genera (*Pseudoxanthomonas* sp., most closely related to *spadix/helianti* and *Xanthomonas*, most closely related to *maliensis/dyei/bromi*) with >97% identity by 16S ONT. By WGS, it was identified as *Pseudoxanthomonas winnipegensis* (98.91%). At the time the ONT data were analyzed, a Centroid for *P. winnipegensis* was not included in the Centroid database (v.r144u311), but it was included in August 2023 (v.r144u553). When re-running the analysis for this sample against the latest 16S Centroid database, *P. winnipegensis* was the best match obtained with a 99.66% identity, followed by *P. helianthi* and *P. spadix*, with 99.64% and 98.08% identities, respectively, for 16S ONT. When re-analyzing the 16S SS data, the best match was also *P. winnipegensis* (99.01%), followed by *Stenotrophomonas tumulicola* (96.81%).

**TABLE 3:** Whole-genome sequencing results for bacterial isolates that are difficult to identify

16S ss	Identity (%)	Match length	Sequence length	16S ONT	Identity (%)	Match length	Sequence length	WGS (% assembly matching to species-level ID)
*Desulfovibrio desulfuricans* *Desulfovibrio legallii*	94.85 93.86	505	1,557 1,443	*Desulfovibrio desulfuricans* *Desulfovibrio legallii*	96.83 95.89	1,515 1,437	1,557 1,443	*Desulfovibrio fairfieldensis* (99.28%)
*Stenotrophomonas tumulicola* *Stenotrophomonas acidaminiphila*	96.81 96.43	502 504	1,472 1,542	*Pseudoxanthomonas spadix* *Pseudoxanthomonas helianthi* *Xanthomonas maliensis* *Xanthomonas dyei* *Xanthomonas bromi*	98.08 97.93 97.17 97.21 97.14	1,512 1,495 1,411 1,468 1,432	1,553 1,494 1,409 1,465 1,429	*Pseudoxanthomonas winnipegensis* (98.91%)
*Anaerococcus octavius*	95.22	481	1,407	*Anaerococcus pacaensis* *Anaerococcus prevencensis*	96.97 96.96	1,288 1,382	1,289 1,378	*Anaerococcus* sp. Marseille (8.15%) Unable to assign definitive bacterial species
*Peptoniphilus stercorisuis*	88.08	478	1,481	*Miniphocaeibacter massiliensis*	91.06	1,398	1,420	*Clostridium botulinum* (29.60%) Unable to assign definitive bacterial species
*Haematobacter massiliensis*	96.67	541	1,471	*Xinfangfangia soli*	96.86	1,431	1,428	*Paracoccus* sp. (51.99%) Unable to assign definitive bacterial species
*Nocardia vaccinii* *Nocardia miyunensis* *Nocardia africana* *Nocardia cerradoensis*	97.70 97.49 97.29 97.08			*Nocardia cerradoensis* *Nocardia africana* *Nocardia araoensis* *Nocardia concava* *Nocardia mikamii*	97.40 97.32 97.23 97.20 97.20	1,462 1,495 1,444 1,466 1,462	1,462 1,492 1,441 1,472 1,466	*Nocardia seriolae* (33.29%) Unable to assign definitive bacterial species

The average nucleotide identities for the other four isolates to the best-matched genomes were less than 52%, suggesting that they were all likely novel bacterial species or that the WGS reference database did not contain whole genomes for these organisms, and a definite bacterial species could not be assigned for any of these isolates.

### R10.3 and R10.4.1 flow cell performance comparison

During the course of this study, ONT released the R10.4 and R10.4.1 flow cells, which showed improved base calling and a significant improvement in the quality of the sequencing data compared to earlier versions (20). The improvement in assembly accuracy shown by the R10.4 flow cells is largely due to enhanced long homopolymer calling, which is generally uncommon in 16S rRNA gene sequences (21, 22). To compare the performance of the new R10.4.1 flow cells with that of the R10.3 flow cells used in this study for bacterial identification, we selected a subset of 44 isolates, re-extracted DNA, and re-sequenced them using the new technology. For 32 of the 44 samples (73%), neither the best-matched species nor the level of identification changed. There was only one sample for which the level of identification increased from *Streptomyces* spp. (*Streptomyces diastaticus* [99.80%] followed by *Streptomyces intermedius* [99.66%]) when using the R10.3 flow cells to *Streptomyces intermedius* (99.66%) when using the R10.4.1 flow cells. However, this improvement was not due to a change in the consensus sequences (which were identical except for two gaps on the R10.3 sequence), but rather their length (1,511 bp for the R10.3 vs. 1,517 bp for the R10.4.1 flow cells), which resulted in an increase in the match length of the R10.4.1 flow cell consensus sequence, with the sequence of S*. diastaticus* decreasing the percent identity to 98.88%. Seven samples showed a slight increase in the percent of identification. For six of these samples, that did not result in a change in the level of identification or the top matched species. For one sample, the best-matched species changed from *Erwinia aphidicola* (99.73%) and *Erwinia persicina* (99.41%) to *Erwinia persicina* (99.87%) and *Erwinia aphidicola* (99.73%). This change was not due to changes in the consensus sequences (which had 100% identity) but rather to the R10.4.1 flow cells providing a shorter sequencing length (1530 bp vs. 1540 bp). For four samples, the percent identification slightly decreased, but the interpretation did not change. There was one *Mycobacterium* isolate for which the top five species identified when sequenced using the R10.3 flow cells completely differed from the ones obtained when sequencing using the R10.4.1 flow cells. However, the interpretation did not change, and it remained as *Mycobacterium* spp. (Table S13). Overall, the new chemistry did not improve the consensus sequences and provided similar results when comparing to the old chemistry with the interpretation of the identification of 42 (95.5%) of the 44 isolates not changing when using the updated flow cells and sequencing chemistry.

### Sample-to-report time and costs

The hands-on time spent from bacterial isolation to generation of data was similar for both methods used at about 2 h. DNA extraction was more time consuming for 16S ONT because the boiled prep used for 16S SS was not suitable for this test, and therefore, manual extraction of DNA was performed in batches of 24 samples. Without considering the time for DNA extraction, it took between 7 h 45 min (for 4 samples) and 17 h 45 m (for 24 samples) for the 16S SS to generate sequencing data. 16S ONT required 4.5 h to prepare the library and an average of 2 h 12 m to generate the 500 reads required for identification. 16S SS sequencing data analysis using the Centroid database took about 15 min per sample (6 h for 24 samples), while 16S ONT data analysis took 1.5 h for 24 samples.

The cost per test from DNA extraction to sequencing data generation was US$25.30 for 16S ONT when multiplexing 24 samples per run (22 samples and 2 controls), while it was US$74 per sample for 16S SS (Table S14).

## DISCUSSION

16S rRNA gene sequencing is frequently used in clinical microbiology laboratories to confirm the identification of cultured bacterial isolates that cannot be identified by MALDI-TOF MS. In this study, we compared the performances of sequencing the first ~500 bp of the 16S rRNA gene by Sanger sequencing vs. sequencing the full length of the gene by ONT for the identification of 153 bacterial clinical isolates within 90 different genera.

Consistent with previous studies (1), the identification accuracy of the full 1,500 bp sequence of the 16S rRNA gene was high when compared to the first 500 bp sequence, with 76% (117 of 153) of the samples showing the same level of identification with the two methods. Moreover, consistent with previous guidelines and studies (4, 23, 24), the 16S ONT workflow, which sequenced the full-length 16S rRNA gene, resulted in an increased level of identification for 28 (18%) isolates when compared to the 16S SS workflow, with a statistically significant increase in the number of bacterial isolates identified at the species level in the challenge group and at the genus level in the prospective group of isolates. Each method used in this study had some constraints. 16S SS covered only ~500 bp of the 16S rRNA gene and is limited to a sequencing depth of one, and 16S ONT has historically been shown to have a low base-calling accuracy, which could hinder its ability to distinguish between highly similar sequences (1). However, since the SmartGene 16S Identification App creates a consensus sequence using all the reads in a sample using a genus-specific profile, the sequencing error in individual sequences must have been minimized, improving the diagnostic accuracy of this method compared to 16S SS. In future studies, the use of the Q20+ sequencing technology can potentially result in an even higher sequencing accuracy. This technology combines the most recent R10.4 chip, which has a higher sequencing accuracy due to its ability to identify homopolymer repeats, with the latest Q20+ chemical reagent. In Q20+ mode, numerous copies of a specific DNA or RNA segment are aligned, which helps to average out random errors and produces a more reliable consensus sequence (5).

Nonetheless, there were still eight species (*Moraxella canis*, *Nocardia kruczakiae*, *Dietzia maris*, *Rhodococcus kroppenstedtii*, *Streptomyces diastaticus*, *Mycobacterium phocaicum*/*mucogenicum*, *Mycobacterium lentiflavum*, and *Gulosibacter* sp.) in which sequencing the entire 16S rRNA gene by 16S ONT resulted in a lower level of identification compared to the sequencing results from the first 500 bp of the gene by 16S SS (Table 1; Table S3). Per CLSI MM18 A2 guidelines, to obtain species-level identification when sequencing the first 500 bp of the 16S rRNA gene, aerobic actinomycetes should show ≥99.6% identity with ≥0.4% separation between different species, and for *Mycobacterium* spp., 100% identity (4). However, whether these guidelines can be extrapolated as such when sequencing the full length of the 16S rRNA gene or whether they should be adapted to the longer sequence has not been determined. It is possible that in some instances the initial 500 bp sequence can provide a better identification than the full-length gene sequence. However, sequencing only 500 bp results in a greater percentage difference between strains, as this region exhibits slightly more diversity per kilobase sequenced, and it may overestimate bacterial diversity when compared with the full-length bacterial 16S rRNA gene. Therefore, a smaller percentage difference between species may be acceptable when sequencing the full length of the 16S rRNA gene (25, 26). Additionally, although 100% identity is mandatory for the identification of *Mycobacterium* spp. per CLSI MM18 A2 guidelines, one or very few mismatches at positions other than the first 500 bp of the 16S rRNA gene may be acceptable for species identification, but an actual threshold has not been established (4). In this study, the application of the modified CLSI guidelines for the interpretation of the 16S ONT sequencing results from aerobic actinomycetes resulted in a higher percentage of organisms identified at the species level than when applying the CLSI MM18 A2 guidelines. The identification of *Mycobacterium* spp. could have also improved if 100% identity was not required for species-level identification when the full-length 16S rRNA gene was sequenced. Unfortunately, WGS was not able to provide a definitive bacterial taxon for some of these organisms, and therefore, additional studies are needed to determine whether the CLSI MM18 A2 guidelines should be modified or updated for the interpretation of full-length 16S rRNA gene sequencing results.

Whole-genome sequencing was performed to identify the definite bacterial taxa for 6 samples difficult to identify and 16 samples with discordant species-level identification by both methods. Nevertheless, most of the isolates difficult to identify showed low query coverage, suggesting they could be novel species. For the samples with discordant results, many of the top species identified by 16S rRNA sequencing did not have representative sequence data in the WGS database. One sample was identified as *Desulfovibrio fairfieldensis* by WGS. However, this organism is not present in the Centroid database because it is not a validly published species (27). *Pseudoxanthomonas winnipegensis* had not been identified at the species level when first analyzed using the Centroid database, but it achieved species-level identification when re-analyzed using an updated version of the database. Achievable matched similarity depends on the adequate representation of the species in the reference database used for analysis. If a species is missing in the database, a sample sequence will yield an inconclusive result. This highlights the limitations of sequencing analysis and the importance of maintaining and frequently updating reference databases. Ideally, reference databases should contain sequences representing the diversity of a taxon, such as the Centroid sequences used in this study. Sequences should be curated, complete, unambiguous nucleotide sequences, with the correct taxonomic nomenclature assigned and validly published under the rules of the International Code of Nomenclature of Prokaryotes (24, 27, 28).

In this study, we performed 16S ONT sequencing for 72 h generating an average of 20,029 reads per sample. However, this workflow is not compatible with clinical microbiology laboratories where timely identification of bacterial pathogens is crucial for diagnosing acute infectious diseases. Nevertheless, based on our analysis, 500 reads would have been sufficient to obtain a reliable identification for most species included in this study, which were obtained in less than 2 h of sequencing in 91% of the cases. Several other studies have shown that 2 h of 16S ONT sequencing and 500 reads is enough for reliable identification even when analyzing mixed bacterial populations or clinical specimens (7, 29–32). However, some bacteria, such as actinomycetes or mycobacteria, may require longer sequencing times as they exhibited the most modest read counts. Several inherent properties of these two groups of bacteria could explain this. Their cell wall composition, with high levels of mycolic acids in mycobacteria and hydrophobic substances in aerobic actinomycetes, makes them more difficult to lyse and extract high quality DNA from (33, 34). DNA fragmentation can occur during extraction because they are more difficult to break open, and if DNA is fragmented during extraction, it can result in shorter DNA fragments which are poorly processed during Nanopore sequencing (35). Also, both bacteria have high guanine-cytosine content in their genomes, which can lead to secondary structures in the DNA that are hard to sequence, especially in longer reads (36).

Repeating sequencing from the same DNA extracts in samples with many mismatches did not seem to improve identification. However, re-sequencing a sample with <100 reads resulted in improved identification of a *Mycobacterium* sp., suggesting that increasing the sequencing depth of 16S ONT with more reads and creating a consensus sequence can reduce the impact of its sequencing error rate.

Without including the time to perform DNA extraction, although the 16S ONT workflow did not reduce the hands-on time, multiplexing 24 samples resulted in a much shorter turnaround time (8 h 12 m) than the 16S SS workflow (23 h 45 min), and the cost per sample was more than three times lower than that of the 16S SS.

There are some limitations to our study. First, Sanger sequencing and Nanopore sequencing rely on different output formats, making a direct comparison challenging; therefore, a consensus sequence was generated from the Nanopore read sequences for comparison purposes. Second, we compared a 500 nt method (16S SS) vs. a ~1,500 nt method (16S ONT). However, the performance of Sanger sequencing decreases with longer sequence reads due to an inability to distinguish single-base pair differences in longer segments (e.g., >900 bp), and therefore, it could have less accuracy in sequencing the full-length 16S rRNA gene (37, 38). Third, we were unable to resolve the discordant results between the two methods because of incomplete whole-genome sequencing data in the reference databases for many of the top species identified by 16S rRNA sequencing. Thus, the final data analysis is a comparison between 16S SS and 16S ONT based on CLSI MM18 2A interpretation guidelines. Fourth, the lack of interpretation guidelines specifically designed for the sequencing of the full 16S rRNA may have resulted in the wrong interpretation of some of the 16S ONT sequencing results. Last, newer ONT sequencing technology (R10.4.1 flow cells) is available with improved per base accuracy that may further reduce the number of reads and sequencing run time required for reliable bacterial isolate identification by 16S ONT. However, our small comparative evaluation did not show significant differences in the clinical performance of the new technology compared to the R10.3 flow cells used in this study, though larger studies may be needed. Regardless, this study provides the basis of a validated end-to-end solution to perform 16S ONT for bacterial identification in clinical and public health laboratories.

### Conclusions

In this study, we compared sequencing of the first 500 bp of the 16S rRNA gene by Sanger sequencing and the full-length 16S rRNA sequencing by ONT for bacterial identification. Overall, the performance of the 16S ONT sequencing was comparable to that of the 16S SS. 16S ONT provided superior resolution for unidentifiable bacterial pathogens, particularly within the gram-negative bacilli and anaerobes, and improved species-level identification compared to 16S SS of the first 500 bp of the gene while significantly reducing the turnaround time and cost. Since the CLSI MM18 2A interpretation guidelines are limited to Sanger-based sequencing of the first 500 bp of the 16S rRNA gene, the development of sequencing interpretation guidelines for results obtained when sequencing the full-length 16S rRNA gene using next-generation sequencing platforms is urgently needed. There also remains a need for open access to comprehensive curated databases to confidently evaluate the results obtained using this method in the clinical microbiology laboratory.

## Data Availability

16S rRNA sequencing data generated can be found in Table S12. Whole-genome sequencing data were deposited to the Sequence Read Archive under http://www.ncbi.nlm.nih.gov/bioproject/1173255 (accession no. PRJNA1173255).

## SUPPLEMENTAL MATERIAL

Supplemental tables jcm.01670-24-s0001.docx Tables S2 to S11, S13, and S14.

Fig. S1 jcm.01670-24-s0002.jpg DNA concentration vs. number of reads.

Fig. S2 jcm.01670-24-s0003.png Number of reads per bacterial group.

Fig. S3 jcm.01670-24-s0004.png Level of identification for aerobic actinomycetes.

Fig. S4 jcm.01670-24-s0005.jpg Differences in consensus based on the number of reads.

Supplemental legends jcm.01670-24-s0006.docx Legends for Fig. S1 to S4.

Table S1 jcm.01670-24-s0007.xlsx Sequencing results.

Table S12 jcm.01670-24-s0008.xlsx Nanopore consensus sequences.

ASM does not own the copyrights to Supplemental Material that may be linked to, or accessed through, an article. The authors have granted ASM a non-exclusive, world-wide license to publish the Supplemental Material files. Please contact the corresponding author directly for reuse.
