# Supplementary material for: Evaluation of long-read 16S rRNA next-generation sequencing for identification of bacterial isolates in a clinical diagnostic laboratory
Source: J Clin Microbiol. 2025 Apr 22;63(5):e01670-24. doi: 10.1128/jcm.01670-24 (PMC12077174; doi:10.1128/jcm.01670-24)
Supplement: Supplemental tables — Tables S2 to S11, S13, and S14. [file jcm.01670-24-s0001.docx]

**Table S2. Results obtained after resequencing isolates with 15 to 50 mismatches on their first 16S ONT sequencing run.**

| **16S ONT identification** | **Sequencing run** | **Percent identification** | **Mismatches** | **Match length** | **Group size** | **Sequence length** | **Reads** |
| --- | --- | --- | --- | --- | --- | --- | --- |
| *Anaerococcus octavius* | 1 | 97.23% | 39 | 1407 | 2 | 1407 | 169095 |
|  | 2 | 97.23% | 39 | 1407 |  |  | 48000 |
| *Anaerococcus octavius* | 1 | 97.73% | 32 | 1409 | 2 | 1407 | 128518 |
|  | 2 | 97.73% | 32 | 1409 |  |  | 24000 |
| *Anaerococcus provincensis* | 1 | 96.88% | 43 | 1380 | 1 | 1378 | 300523 |
|  | 2 | 96.89% | 43 | 1382 |  |  | 40000 |
| *Brevibacillus formosus* | 1 | 98.65% | 20 | 1482 | 78 | 1486 | 11998 |
|  | 2 | 98.65% | 20 | 1477 |  |  | 28000 |
| *Corynebacterium pilbarense* | 1 | 98.85% | 16 | 1393 | 7 | 1392 | 3511 |
|  | 2 | 98.85% | 16 | 1393 |  |  | 4000 |
| *Desulfovibrio intestinalis* | 1 | 98.34% | 25 | 1502 | 4 | 1509 | 9900 |
|  | 2 | 98.33% | 25 | 1496 |  |  | 16000 |
| *Gulosibacter chungangensis* | 1 | 96.55% | 50 | 1451 | 3 | 1451 | 15159 |
|  | 2 | 96.55% | 50 | 1451 |  |  | 12000 |
| *Gulosibacter chungangensis* | 1 | 96.55% | 50 | 1451 | 3 | 1451 | 4842 |
|  | 2 | 96.55% | 50 | 1451 |  |  | 16000 |
| *Massilia aurea* | 1 | 97.89% | 31 | 1466 | 52 | 1463 | 11999 |
|  | 2 | 97.95% | 30 | 1466 |  |  | 32000 |
| *Mixta gaviniae* | 1 | 98.28% | 26 | 1514 | 35 | 1543 | 27729 |
|  | 2 | 98.41% | 24 | 1512 | 35 | 1543 | 64000 |
| *Mixta tenebrionis* | 1 | 97.82% | 31 | 1420 | 1 | 1412 | 1808 |
|  | 2 | 97.82% | 31 | 1420 |  |  | 12000 |
| *Moraxella porci* | 1 | 98.83% | 16 | 1364 | 4 | 1367 | 8289 |
|  | 2 | 98.83% | 16 | 1364 |  |  | 36000 |
| *Myroides pelagicus* | 1 | 97.16% | 42 | 1477 | 6 | 1486 | 19111 |
|  | 2 | 97.16% | 42 | 1477 | 125 | 1491 | 32000 |
| *Pseudoxanthomonas helianthi* | 1 | 98.26% | 26 | 1492 | 3 | 1494 | 11684 |
|  | 2 | 98.26% | 26 | 1492 |  |  | 28000 |
| *Roseomonas musae* | 1 | 98.03% | 28 | 1422 | 2 | 1422 | 13126 |
|  | 2 | 98.03% | 28 | 1422 |  |  | 44000 |
| *Schaalia odontolytica* | 1 | 97.88% | 32 | 1506 | 110 | 1544 | 5369 |
|  | 2 | 97.87% | 32 | 1505 |  |  | 8000 |
| *Corynebacterium fournieri* | 1 | 99.19% | *12* | *1482* | *1* | *1481* | *7669* |
|  | 2 | 99.19% | *12* | *1481* |  |  | 3882 |

**Table S3. Results obtained after resequencing isolates with less than 1000 reads on their first 16S ONT sequencing.**

| **16S ONT identification** | **Sequencing run** | **Percent identification** | **Mis-matches** | **Match length** | **Group size** | **Sequence length** | **Reads** |
| --- | --- | --- | --- | --- | --- | --- | --- |
| *Mycobacterium paragordonae*  *Mycobacterium gordonae* | 1 | 99.00%  98.86% | 15  17 | 1493 | 12  125 | 1534  1534 | 68 |
| *Mycobacterium gordonae*  *Mycobacterium paragordonae* | 2 | 99.12%  99.00% | 13  15 | 1485  1493 | 125  12 | 1491  1534 | 920 |
| *Brucella intermedia* | 1 | 99.03% | 14 | 1449 | 399 | 1479 | 212 |
|  | 2 | 99.03% | 14 | 1449 |  |  | 4000 |
| *Corynebacterium fournieri* | 1 | 99.19% | 12 | 1482 | 1 | 1481  1481 | 769 |
|  | 2 | 99.19% | 12 | 1481 |  |  | 3882 |
| *Schaalia cardifensis* | 1 | 99.01% | 15 | 1514 | 12 | 1556  1556 | 576 |
|  | 2 | 99.01% | 15 | 1516 |  |  | 2326 |
| *Nocardia asiatica* | 1 | 99.40% | 9 | 1500 | 34 | 1505 | 714 |
|  | 2 | n/a | n/a |  |  |  | 13 |
| *Nocardia brasiliensis* | 1 | 99.53% | 7 | 1486 | 142 | 1524  1524 | 906 |
|  | 2 | 99.54% | 7 | 1515 |  |  | 455 |

**Table S4. Correlation between 16S SS and 16S ONT sequencing results for prospective isolates following CLSI MM18 A2 guidelines for interpretation (n=102)**

|  | | **Identification by 16S SS** | | | |
| --- | --- | --- | --- | --- | --- |
|  |  | **No ID**  **(n=5 – 5%)** | **Family**  **(n=10 - 10%)** | **Genus**  **(n=22 – 22%)** | **Species (n=65 – 64%)** |
| **Identification by 16S ONT** | **No ID**  **(n=1 – 1%)** | *1*  *Anaerobic Gram-positive cocci* |  |  |  |
|  | **Family**  **(n=6 – 6%)** | *2*  *Peptoniphilaceae*  *Desulfovibrionaceae* | *4*  *Peptoniphilaceae*  *Paracoccaceae*  *Microbacteriaceae*  *Nocardiaceae* |  |  |
|  | **Genus**  **(n=30 – 29%)** | *2*  *Anaerococcus*  *Mixta* | *6*  *Myroides Pseudoxanthomonas (x3)*  *Schaalia*  *Nocardia* | *14*  *Corynebacterium*  *Brevibacillus*  *Massilia*  *Roseomonas*  *Porphyromonas*  *Anaerococcus*  *Mixta*  *Mycobacterium (x5)*  *Actinomadura*  *Gordonia* | *8*  *Moraxella canis*  *Nocardia kruczakiae Mycobacterium phocaicum/mucogenimum Mycobacterium lentiflavum*  *Dietzia maris*  *Rhodococcus kroppenstedtii (x2)*  *Streptomyces diastaticus* |
|  | **Species**  **(n=65– 64%)** |  |  | 8  *Lysinibacillus boronitolerans*  *Bosea robiniae*  *Pseudoxanthomonas kaohsiungensis*  *Cosenzaea myxofasciens*  *Varibaculum cambriense*  *Paenibacillus selenitireducens Mycobacterium arcueilense Mycobacterium gastri* | 57 |

**Table S5. Correlation between 16S SS and 16S ONT sequencing results for challenge isolates following CLSI MM18 A2 guidelines for interpretation (n=51)**

|  | | **Identification by 16S SS** | | |
| --- | --- | --- | --- | --- |
|  |  | **No ID**  **(n=3 – 6%)** | **Genus**  **(n=10 – 20%)** | **Species**  **(n=38 – 75%)** |
| **Identification by 16S ONT** | **Family**  **(n=1 - 2%)** |  | *1 Gulosibacter* |  |
|  | **Genus**  **(n=4 – 8%)** | *2*  *Butyricimonas*  *Desulfovibrio* | *Nocardia (x2)* |  |
|  | **Species**  **(n=46 – 90%)** | *1*  *Brucella intermedia* | *7*  *Aggregatibacter actinomycetemcomitans*  *Brevibacterium paucivorans*  *Klebsiella grimontii*^a^  *Cellulomonas pakistanensis*  *Edwardsiella tarda*  *Peptoniphilus asaccharolyticus*  *Slackia exigua* | *38* |

^a^ Species-level ID of *Klebsiella grimontii* would be reported as part of the *Klebseilla oxytoca* complex as 16S rRNA gene sequencing does not discern between members of this complex

**Table S6. Correlation between 16S SS and 16S ONT sequencing results for prospective and challenge aerobic actinomycetes following CLSI MM18 2A guidelines for interpretation (n=15) and comparison with the results obtained using the modified CLSI interpretation guidelines. The number of isolates and percentages shown in each category are from the CLSI MM18 2A interpretation.**

| **Aerobic Actinomycetes** | | **Identification by 16S SS** | | | | | |
| --- | --- | --- | --- | --- | --- | --- | --- |
|  |  | **Family**  **(n=2 - 13%)** | **Genus** **(n=4 – 27%)** | | **Species (n=9 - 60%)** | | |
|  |  |  | **CLSI MM18 2A** | **Modified CLSI** | **CLSI MM18 2A** | | **Modified CLSI** |
| **Identification by 16S ONT** | **Family (n=1 - 7%)** | 1  *Nocardiaceae* |  | |  | | |
|  | **Genus** **(n=9 - 60%)** | 1  *Nocardia* | 4 | | 4 | | |
|  |  |  | *Actinomadura* sp. | *Actinomadura geliboluensis/cremea* by both methods | *Nocardia kruczakiae* | *Nocardia kruczakiae* by 16S SS and *Nocardia* sp. by 16S ONT | |
|  |  |  | *Nocardia* sp. | *Nocardia brasiliensis* by 16S SS and *Nocardia* sp. by 16S ONT | *Dietzia maris* | *Dietzia maris* by 16S SS and *Dietzia kunjamensis* by 16S ONT | |
|  |  |  | *Nocardia* sp. | *Nocardia* sp. by both methods | *Rhodococcus kroppenstedtii (x2)* | *Rhodococcus kroppenstedtii* by 16S SS and *Rhodococcus corynebacteroides* by 16S ONT | |
|  |  |  | *Gordonia* sp. | *Gordonia sp*. by 16S SS and *Gordonia lacunae/ terra/*  *honkongnensis* by 16S ONT |  |  |  |
|  | **Species**  **(n=5 - 33%)** |  |  | | 5  *Nocardia nova*  *Nocardia thailandica*  *Gordonia otitidis*  *Dietzia aurantica*  *Streptomyces diasticus* | | |

**Table S7. Correlation between 16S SS and 16S ONT sequencing results for *Mycobacterium* spp. following CLSI MM18 A2 guidelines for interpretation (n=9)**

| ***Mycobacterium* spp. (n=9)** | | **Identification by 16S SS** | |
| --- | --- | --- | --- |
|  |  | **Genus-level**  **(n=7 – 78%)** | **Species**  **(n=2 – 22%)** |
| **Identification by 16S ONT** | **Genus-level**  **(n=7 – 78%)** | 5 | 2  *M. phocaicum/mucogenicum*  *M. lentiflavum* |
|  | **Species-level**  **(n=2 – 22%)** | 2  *M. arcuilense*  *M. gastri* |  |

**Table S8. Correlation between 16S SS and 16S ONT sequencing results for prospective and challenge coryneform Gram-positive rods following CLSI MM18 A2 guidelines for interpretation (n=12).**

| **Coryneform GPR – n=12** | | **Identification by 16S SS** | | |
| --- | --- | --- | --- | --- |
|  |  | **Family-level**  **(n=1 – 9%)** | **Genus-level**  **(n=4 – 33%)** | **Species level**  **(n=7 – 58%)** |
| **Identification by 16S ONT** | **Family**  **(n=2 – 16%)** | *1*  *Microbacteriaceae* | *1*  *Gulosibacter* |  |
|  | **Genus ID**  **(n=1 – 9%)** |  | *1*  *Corynebacterium* |  |
|  | **Species ID**  **(n=9 – 75%)** |  | *2*  *Brevibacterium paucivorans*  *Cellulomonas hominis* | 7 |

**Table S9. Correlation between 16S SS and 16S ONT sequencing results for prospective and challenge Gram-negative bacilli following CLSI MM18 A2 guidelines for interpretation (n61)**

| **Gram negative rods**  **n=61** | | **Identification by 16S SS** | | | |
| --- | --- | --- | --- | --- | --- |
|  |  | **Unable to ID**  **(n=4 – 7%)** | **Family**  **(n=5 – 8%)** | **Genus-level**  **(n=9 – 15%)** | **Species**  **(n=45 – 70%)** |
| **Identification by 16S ONT** | **Family-level**  **(n= 1 – 2%)** |  | *1*  *Paracoccaceae* |  |  |
|  | **Genus-level**  **(n=9 – 15%)** | *1*  *Mixta* | *4*  *Pseudoxanthomonas (x3)*  *Myroides* | *3*  *Massilia*  *Mixta*  *Roseomonas* | *1 Moraxella canis* |
|  | **Species-level**  **(n=51 – 84%)** | 1  *Brucella intermedia* |  | *6*  *Aggregatibacter actinomycetemcomitans Edwardsiella tarda*  *Klebsiella grimontii*^a^  *Bosea roniniae Pseudoxanthomonas kaohsiungensis*  *Cosenzaea myxofasciens* | *44* |

^a^ Species-level ID of *Klebsiella grimontii* would be reported as part of the *Klebseilla oxytoca* complex as 16S rRNA gene does not discern members of this complex

**Table S10. Correlation between 16S SS and 16S ONT sequencing results for prospective and challenge anaerobes following CLSI MM18 A2 guidelines for interpretation (n=38).**

| **Anaerobes n=38** | | **Identification by 16S SS** | | | |
| --- | --- | --- | --- | --- | --- |
|  |  | **Unable to ID**  **(n=6 – 16%)** | **Family-level**  **(n=2 – 5%)** | **Genus-level**  **(n=5 – 13%)** | **Species level (n=25 – 66%)** |
| **Identification by 16S ONT** | **No ID**  **(n=1 – 3%)** | *1* |  |  |  |
|  | **Family-level**  **(n=3 – 8%)** | *2 Peptoniphilaceae*  *Desulfovibrionaceae* | *1 Peptoniphilaceae* |  |  |
|  | **Genus-level**  **(n=6 – 16%)** | *3*  *Anaerococcus,*  Butyricimonas,  Desulfovibrio | *1 Schaalia* | *2*  *Anaerococcus*  *Porphyromonas* |  |
|  | **Species-level (n=28 – 74%)** |  |  | *3*  *Varibaculum cambriense*  *Peptoniphilus asaccharolyticus*  *Slackia exigua* | *25* |

**Table S11. Isolates that took more than 2 hours to reach 500 reads.**

| **Time to 500 reads** | **Total number of reads** | **Consensus with 500 reads vs. total reads** | **Identification by 16S ONT** |
| --- | --- | --- | --- |
| 0 days 03:25:02 | 1808 | Different | *Mixta tenebrionis (97.82%) vs Mixta tenebrionis (97.75%)* |
| 0 days 04:46:55 | 1958 | Same | *Gordonia otitidis (100%)* |
| 0 days 04:52:05 | 1817 | Same | *Actinomadura geliboluensis (99.73%)* |
| 0 days 06:29:24 | 1305 | Same | *Actinomyces israelii (99.67%)* |
| 0 days 06:41:59 | 1186 | Same | *Turicibacter sanguinis* (100%) |
| 0 days 09:18:57 | 977 | Same | *Bilophila wadsworthia (99.93%)* |
| 0 days 10:58:51 | 1461 | Same | *Leuconostoc mesenteroides (99.74%)* |
| 0 days 15:13:40 | 1021 | Same | *Streptomyces diastaticus (99.80%)* |
| 0 days 15:28:10 | 753 | Same | *Dietzia kunjamensis (99.93%)* |
| 1 day 19:18:52 | 576 | Same | *Schaalia cardiffensis (99.01%)* |
| 2 days, 3:10:01 | 569 | Same | *Nocardia thailandica (100%)* |
| 2 days 10:50:09 | 1544 | Same | *Eggerthella lenta* (99.80%) |

**Table S13. Sequencing results for a subset of 44 samples comparing the performance of R10.3 and 10.4.1 flowcells for clinical bacterial identification.**

| **Sanger Identification** | **ONT R10.3 Identification** | **ONT 10.4.1 Identification** |
| --- | --- | --- |
| *Peptoniphilus stercorisuis (88.08%) Peptoniphilis methioninivorax (88.14%) Anaerosphaera aminiphila (86.19%)* | *Miniphocaeibacter massiliensis (91.06%) Anaerosphera multitolerans (87.01%)* | *Miniphocaeibacter halophilus (91.04%) Miniphocaeibacter massiliensis (91.96%* |
| *Schaalia odontolytica (96.46%)* | *Schaalia odontolytica (97.88%) Schaalia meyeri (96.70%)* | *Schaalia odontolytica (97.88%) Schaalia meyeri (96.70%)* |
| *Ezakiella massiliensis (99.42%) Ezakiella peruensis (99.59%)* | *Ezakiella massiliensis (99.80%) Ezakiella peruensis (98.18%)* | *Ezakiella massiliensis (99.80%) Ezakiella peruensis (98.18%)* |
| *Anaerococcus octavius (95.22%)* | *Anaerococcus pacaensis (96.97%) Anaerococcus provencensis (96.96%)* | *Anaerococcus pacaensis (96.97%) Anaerococcus provencensis (96.96%)* |
| *Alistides onderdonkii (99.60%)* | *Alistipes onderdonkii (99.80%) Alistipes finegoldii (97.94%)* | *Alistipes onderdonkii (99.80%) Alistipes finegoldii (96.91%)* |
| *Fannyhessea vaginae (99.61%)* | *Fannyhessea vaginae (99.73%) Olsenella prefusa (94.18%)* | *Fannyhessea vaginae (99.80%) Olsenella prefusa (94.18%)* |
| *Lysinibacillus macroides (98.28%) Lysinibacillus xylanilyticus (96.93%) Lysinibacillus sphaericus (96.36%)* | *Lysinibacillus macroides (99.47%) Lysinibacillus boronitolerans (99.44%) Lysinibacillus capsici (99.36%)* | *Lysinibacillus macroides (99.47%) Lysinibacillus boronitolerans (99.44%) Lysinibacillus capsici (99.36%)* |
| *Moraxella canis (99.80%) Moraxella catarrhalis (97.25%) Moraxella porci (98.32%)* | *Moraxella porci (98.83%) Moraxella canis (98.80%) Moraxella caprae (98.32%) Moraxella pluranimalium (97.77%) Moraxella cuniculi (97.33%)* | *Moraxella porci (98.83%) Moraxella canis (98.67%) Moraxella caprae (98.32%) Moraxella pluranimalium (97.77%) Moraxella cuniculi (97.21%)* |
| *Cellulosimicrobium funkei (99.20%) Cellulosimicrobium cellulans (98.80%)* | *Cellulosimicrobium cellulans (99.87%) Cellulosimicrobium funkei (99.73%)* | *Cellulosimicrobium cellulans (99.87%) Cellulosimicrobium funkei (99.73%)* |
| *Pseudomonas mosselii (100%) Pseudomonas asiatica (99.59%) Pseudomonas taiwanensis (99.59%) Pseudomonas entomophila (99.39%) Pseudomonas monteilii (99.39%)* | *Pseudomonas mosselii (99.73%) Pseudomonas juntendi (99.65%) Pseudomonas taiwanensis (99.52%) Pseudomonas sichuanensis (99.34%)* | *Pseudomonas mosselii (99.87%) Pseudomonas xantholysinigenes (99.80%) Pseudomonas fakonensis (99.80%) Pseudomonas muyukensis (99.67%) Pseudomonas entomophila (99.67%)* |
| *Brevundimonas vesicularis (100%) Brevundimonas nasdae (100%) Brevundimonas intermedia (99.56%) Brevundimonas aurantiaca (99.56%)* | *Brevundimonas huaxiensis (99.93%) Brevundimonas nasdae (99.51%) Brevundimonas vesicularis (99.51%) Brevundimonas intermedia (99.37%)* | *Brevundimonas huaxiensis (99.93%) Brevundimonas nasdae (99.51%) Brevundimonas vesicularis (99.51%) Brevundimonas intermedia (99.37%)* |
| *Herbaspirillum frisingense (99.61%) Herbaspirillum seropedicae (98.03%) Herbaspirillum chlorophenolicum (98.21%) Herbaspirillim robinae (97.83%) Herbaspirillum rubrisubalbicans (97.63%) Herbaspirillum aquaticum (97.62%) Herbaspirillum huttiense (97.42%)* | *Herbaspirillum frisingense (99.67%) Herbaspirillum chlorophenolicum (99.31%) Herbaspirillum aquaticum (99.31%) Herbaspirillum huttiense (99.12%)* | *Herbaspirillum frisingense (99.34%) Herbaspirillum chlorophenolicum (99.31%) Herbaspirillum aquaticum (99.31%) Herbaspirillum huttiense (98.79%)* |
| *Erwinia persicina (99.43%) Erwinia billingiae (98.49%) Erwinia rwandensis (98.11%) Erwinia tasmaniensis (97.74%) Erwinia rhapontici (98.12%) Erwinia aphidicola (99.21%)* | *Erwinia aphidicola (99.73%) Erwinia persicina (99.41%)* | *Erwinia persicina (99.87%). Erwinia aphidicola (99.73%) Erwinia rhapontici (99.40%)* |
| *Pseudoglutamicibacter cumminsii (99.58%) Pseudoglutamicibacter albus (98.96%)* | *Pseudoglutamicibacter cumminsii (99.73%) Pseudoglutamicibacter albus (99.39%)* | *Pseudoglutamicibacter cumminsii (99.73%) Pseudoglutamicibacter albus (99.33%)* |
| *Robertmurraya (Bacillus) siralis (99.19%)* | *Bacillus siralis (99.72%) Bacillus purgationiresistens (97.46%)* | *Robertmurraya siralis (99.72%) Cytobacillus purgationiresistens (97.46%)* |
| *Elizabethkingia anophelis (99.80%)* | *Elizabethkingia anophelis (99.80%) Elizabethkingia meningoseptica (98.65%)* | *Elizabethkingia anophelis (99.80%) Elizabethkingia meningoseptica (98.65%)* |
| *Proteus vulgaris (97.68%) Cosenzaea myxofasciens (98.99%)* | *Cosenzaea myxofaciens (99.59%) Proteus penneri (98.43%) Proteus vulgaris (98.31%)* | *Cosenzaea myxofaciens (99.59%) Proteus hauseri (98.64%) Proteus penneri (98.43%)* |
| *Kytococcus schroeteri (99.80%)* | *Kytococcus schroeteri (99.87%) Kytococcus aerolatus (98.94%) Kytococcus sedentarius (97.89%)* | *Kytococcus schroeteri (99.87%) Kytococcus aerolatus (98.94%) Kytococcus sedentarius (97.96%)* |
| *Roseomonas musae (98.92%) Roseomonas aerophila (98.02%)* | *Roseomonas musae (98.03%) Roseomonas aerophila (97.83%)* | *Roseomonas musae (98.03%) Roseomonas aerophila (97.83%)* |
| *Sphingomonas aurantiaca (98.06%) Sphingomonas faeni (98.48%) Sphingomonas olei (99.78%)* | *Sphingomonas olei (99.79%) Sphingomonas mucosissima (99.71%) Sphingomonas panaciterrae (99.14%)* | *Sphingomonas olei (99.79%) Sphingomonas mucosissima (99.71%) Sphingomonas panaciterrae (99.14%)* |
| *Luteibacter yeojuensis (97.14%) Luteibacter rhizovicinus (96.95%) Luteibacter anthropi (99.80%)* | *Luteibacter anthropi (100%) Luteibacter yeojuensis (98.33%) Luteibacter rhizovicinus (98.28%)* | *Luteibacter anthropi (100%) Luteibacter yeojuensis (98.33%) Luteibacter rhizovicinus (98.28%)* |
| *Chryseobacterium takakiae (99.61%) Chryseobacterium hispalense (98.06%)* | *Chryseobacterium takakiae (99.93%) Chryseobacterium profundimaris (99.17%)* | *Chryseobacterium takakiae (99.86%) Chryseobacterium profundimaris (99.17%)* |
| *Streptomyces diastaticus (100%) Streptomyces koyangensis (99.20%)* | *Streptomyces diastaticus (99.80%) Streptomyces intermedius (99.66%)* | Streptomyces intermedius (99.66%) Streptomyces aureoverticillatus (98.92%) |
| *Gordonia terrae (99.41%) Gordonia lacunae (99.40%) Gordonia hongkongensis (99.40%)* | *Gordonia terrae (99.87%) Gordonia lacunae (99.87%) Gordonia hongkongensis (99.87%) Gordonia didemni (99.71%)* | *Gordonia terrae (99.87%) Gordonia lacunae (99.87%) Gordonia hongkongensis (99.87%) Gordonia didemni (99.71%)* |
| *Dietzia maris (98.19%) Dietzia natronolimnaea (97.99%) Dietzia aurantiaca (99.79%)* | *Dietzia aurantiaca (100%) Dietzia aerolata (99.35%)* | *Dietzia aurantiaca (100%) Dietzia aerolata (99.35%)* |
| *Rhodococcus kroppenstedtii (100%) Rhodococcus corynebacteroides (99.58%)* | *Rhodococcus corynebacteroides (99.66%) Rhodococcus kroppenstedtii (99.39%) Rhodococcus trifolii (99.05%)* | *Rhodococcus corynebacteroides (99.53%) Rhodococcus kroppenstedtii (99.39%) Rhodococcus trifolii (99.05%)* |
| *Dietzia maris (99.60%) Dietzia natronolimnacea (98.60%)* | *Dietzia kunjamensis (99.93%) Dietzia maris (99.86%)* | *Dietzia kunjamensis (99.93%) Dietzia maris (99.86%)* |
| *Rhodococcus kroppenstedtii (99.79%) Rhodococcus corynebacteroides (99.37%)* | *Rhodococcus corynebacteroides (99.60%) Rhodococcus kroppenstedtii (99.39%) Rhodococcus trifolii (99.05%)* | *Rhodococcus corynebacteroides (99.66%) Rhodococcus kroppenstedtii (99.39%) Rhodococcus trifolii (99.05%)* |
| *Actinomadura geliboluensis (100%) Actinomadura cremea (100%) Actinomadura roseirufa (99.58%) Actinomadura formosensis (99.17%)* | *Actinomadura geliboluensis (99.73%) Actinomadura cremea (99.73%)* | *Actinomadura geliboluensis (99.87%) Actinomadura cremea (99.87%)* |
| *Nocardia anaemiae (98.58%) Nocardia aurea (98.38%) Nocardia vinacea (98.77%)* | *Nocardia vinacea (99.59%) Nocardia pseudovaccinii (99.03%)* | *Nocardia vinacea (99.59%) Nocardia pseudovaccinii (99.03%)* |
| *Nocardia kruczakiae (100%) Nocardia elegans (99.58%) Nocardia cerradoensis (99.58%) Nocardia mikamii (99.58%) Nocardia veterana (99.58%) Nocardia africana (99.36%)* | *Nocardia elegans (99.59%) Nocardia cerradoensis (99.45%) Nocardia kruczakiae (99.38%) Nocardia mikamii (99.25%) Nocardia africana (99.18%)* | *Nocardia elegans (99.59%) Nocardia cerradoensis (99.45%) Nocardia kruczakiae (99.38%) Nocardia mikamii (99.25%) Nocardia africana (99.06%)* |
| *Nocardia thailandica (100%)* | *Nocardia thailandica (100%) Nocarida asteroides (98.81%)* | *Nocardia thailandica (100%) Nocardia asteroides (98.99%)* |
| *Gordonia otitidis (99.80%) Gordonia sputi (99.19%) Gordonia aichiensis (99.18%)* | *Gordonia otitidis (100%) Gordonia sputi (99.59%) Gordonia aichiensis (99.52%)* | *Gordonia otitidis (100%) Gordonia sputi (99.59%) Gordonia aichiensis (99.52%)* |
| *Mycobacterium septicum (99.80%) Mycobacterium peregrinum (99.60%) Mycobacterium lutetiense (99.60%) Mycobacterium fortuitum (99.20%) Mycobacterium alvei (99.20%)* | *Mycobacterium arcueilense (100%) Mycobacterium peregrinum (99.86%) Mycobacterium septicum (99.66%) Mycobacteriun montmartrense (99.52%) Mycobacterium lutetiense (99.46%)* | *Mycobacterium arcueilense (100%) Mycobacterium peregrinum (99.80%) Mycobacterium septicum (99.59%) Mycobacteriun montmartrense (99.52%) Mycobacterium lutetiense (99.46%)* |
| *Mycobacterium phocaicum (100%) Mycobacterium mucogenicum (100%) Mycobacterium singnathidarum (99.40%) Mycobacterium houstonense (99.60%) Mycobacterium conceptionense (99.60%) Mycobacterium fortuitum (99.60%) Mycobacterium senegalense (99.60%)* | *Mycobacterium phocaicum (99.86%) Mycobacterium mucogenicum (99.86%) Mycobacterium farcinogenes (99.11%) Mycobacterium houstonenese (99.05%)* | *Mycobacterium phocaicum (99.86%) Mycobacterium mucogenicum (99.86%) Mycobacterium houstonenese (99.05%)* |
| *Mycobacterium porcinum (99.80%) Mycobacterium neworleansense (99.80%) Mycobacterium boenickei (99.59%)* | *Mycobacterium porcinum (99.87%) Mycobacterium neworleansense (99.80%) Mycobacterium boenickei (99.73%) Mycobacterium senegalense (99.60%) Mycobacteriun conceptionense (99.60%)* | *Mycobacterium porcinum (99.87%) Mycobacterium neworleansense (99.80%) Mycobacterium boenickei (99.73%) Mycobacterium senegalense (99.60%) Mycobacteriun farcinogenes (99.59%)* |
| *Mycobacterium paragordonae (99.61%) Mycobacterium gordonae (98.82%)* | *Mycobacterium godonae (99.12%) Mycobacterium gordonae (99.00%)* | Mycobacterium gordonae (99.93%) Mycobacterium paragordonae (99.80%) |
| *Mycobacterium paraffinicum (99.59%) Mycobacterium scrofulaceum (99.38%) Mycobacterium mantenii (98.96%)* | *Mycobacterium paraffinicum (99.73%) Mycobacterium scrofulaceum (99.66%) Mycobacterium mantenii (99.32%)* | *Mycobacterium paraffinicum (99.73%) Mycobacterium scrofulaceum (99.66%) Mycobacterium mantenii (99.26%)* |
| *Mycobacterium lentiflavum (99.79%) Mycobacterium palustre (99.17%) Mycobacterium simiae (98.76%)* | *Mycobacterium lentiflavum (99.86%) Mycobacterium simiae (99.46%) Mycobacterium triplex (99.26%) Mycobacterium shigaense (99.19%) Mycobacterium sherrisii (99.19%)* | *Mycobacterium lentiflavum (99.86%) Mycobacterium simiae (99.46%) Mycobacterium triplex (99.26%) Mycobacterium shigaense (99.19%) Mycobacterium sherrisii (99.19%)* |
| *Mycobacterium lentiflavum (100%) Mycobacterium simiae (99.00%) Mycobacterium saskarchewanense (99.00%) Mycobacterium palustre (99.39%)* | *Mycobacterium lentiflavum (99.86%) Mycobacterium simiae (99.46%) Mycobacterium triplex (99.26%) Mycobacterium shigaense (99.19%) Mycobacterium sherrisii (99.19%)* | *Mycobacterium lentiflavum (99.86%) Mycobacterium simiae (99.46%) Mycobacterium triplex (99.26%) Mycobacterium shigaense (99.19%) Mycobacterium sherrisii (99.19%)* |
| *Mycobacterium kansasii (99.61%) Mycobacterium innocens (99.22%) Mycobacterium gastri (99.80%) Mycobacterium persicum (98.26%) Mycobacterium pseudokansasii (98.45%)* | *Mycobacterium gastri (100%) Mycobacterium kansasii (99.87%) Mycobacterium innocens (99.73%) Mycobacterium persicum (99.53%) Mycobacterium pseudokansasii (99.47%)* | Mycobacterium intracellulare (99.80%) Mycobacterium marseillense (99.73%) Mycobacterium timonense (99.66%) Mycobacterium arosiense (99.53%) Mycobacterium vulneris (99.46%) |
| *Mycobacterium abscessus (99.60%) Mycobacterium saopaulense (99.60%) Mycobacterium stephanolepidis (99.60%) Mycobacterium chelonae (99.60%)* | *Mycobacterium abscessus (99.80%) Mycobacterium franklinii (99.79%) Mycobacterium saopaulense (99.73%) Mucobacterium chelonae (99.73%) Mycobacterium stephanolepidis (99.53%)* | *Mycobacterium abscessus (99.80%) Mycobacterium franklinii (99.79%) Mycobacterium saopaulense (99.73%) Mucobacterium chelonae (99.73%) Mycobacterium stephanolepidis (99.53%)* |
| *Schaalia cardifensis (99.22%) Next 92.56%* | *Schaalia cardiffensis (99.01%) Schaalia meyeri (96.28%)* | *Schaalia cardiffensis (99.74%) Schaalia meyeri (95.65%)* |
| Varibaculum cambriense (98.33%) | *Varibaculum cambriense (99.26%) Varibaculum vaginae (98.27%)* | *Varibaculum cambriense (99.26%) Varibaculum vaginae (98.27%)* |

**Table S14. Cost analysis**

| **Cost of 16S ONT (24 samples/run)** | | | | |
| --- | --- | --- | --- | --- |
| **Item Name** | **Cost per unit** | **Runs/Samples** | **Cost per run** | **Cost per sample** |
| **16S Barcoding Kit** | $650 | 6 runs | $650/6=$108.3 | $4.5 |
| **GridION Flow Cell** | $500 | up to 24 samples/run | $500 | $20.8 |
| **Total** |  |  | $608.30 | $25.3 |

| **Cost of Sanger sequencing** | | | |
| --- | --- | --- | --- |
|  | **Cost per unit** | **Unit** | **Cost per sample** |
| **Big Dye terminator** | $1000 | 100 reactions | $20 |
| **MicroCon Centrifugal filters** | $400 | 100 filters | $8 |
| **Qiagen DyeEx Spin kit** | $1148 | 250 columns | $9 |
| **MicroSeq 500 16S rDNA PCR kit** | $1042 | 50 reactions | $21 |
| **MicroSeq 500 16S rDNA sequencing kit** | $612 | 55 reactions | $12 |
| **Cep-C molecular water, gel electrophoresis, reagent grade water, Hi-Diformamide, negative control (PrepMan Ultra), 96 well sequencer microtiter plate** |  |  | $4 |
| **Total cost** |  |  | $74 |

**Figure S1. Correlation between DNA concentration and number of reads obtained for each sequencing run by 16S ONT sequencing.**

**Figure S2. Number of reads obtained after 16S ONT sequencing by bacterial group**

**Figure S3. Level of identification obtained for aerobic actinomycetes by 16S SS and 16S ONT applying CLSI MM18 A2 (CLSI) and modified-CLSI (mCLSI) guidelines for interpretation.**


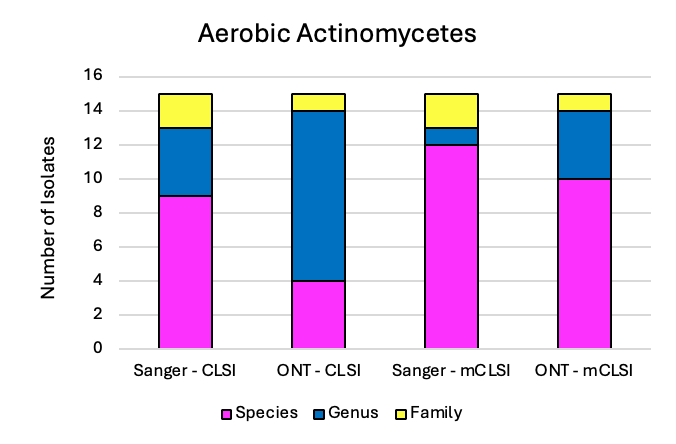


**Figure S4. Evolution of the number of identical consensuses, sum of the differences and number of samples according to the number of reads. Blue line: for 67 samples, the consensus achieved with 6000 reads was the same as the consensus obtained with the total number of reads. Seventeen samples had a different consensus (maximum was 4 nucleotide difference) but none of these differences changed the identification.**

**
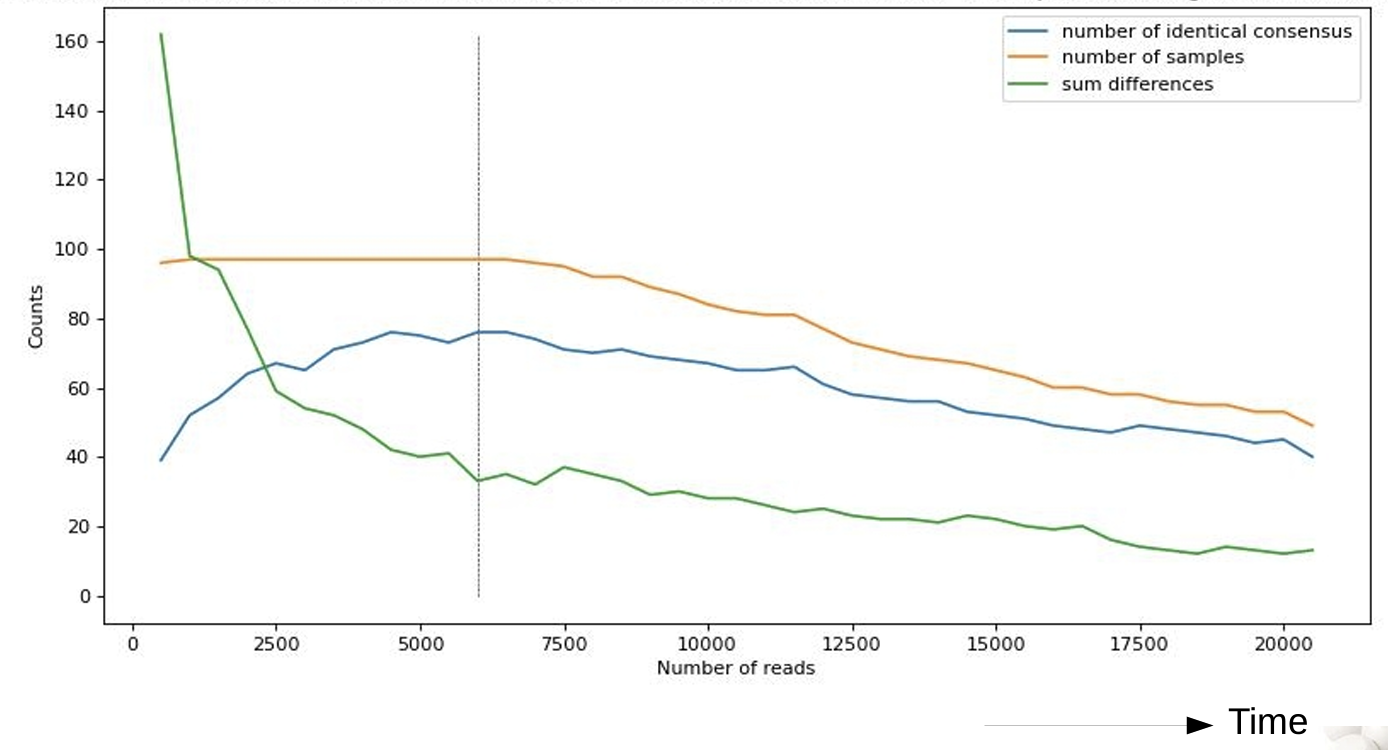
**
