# Supplementary figures and images for: Evaluation of long-read 16S rRNA next-generation sequencing for identification of bacterial isolates in a clinical diagnostic laboratory
Source: J Clin Microbiol. 2025 Apr 22;63(5):e01670-24. doi: 10.1128/jcm.01670-24 (PMC12077174; doi:10.1128/jcm.01670-24)

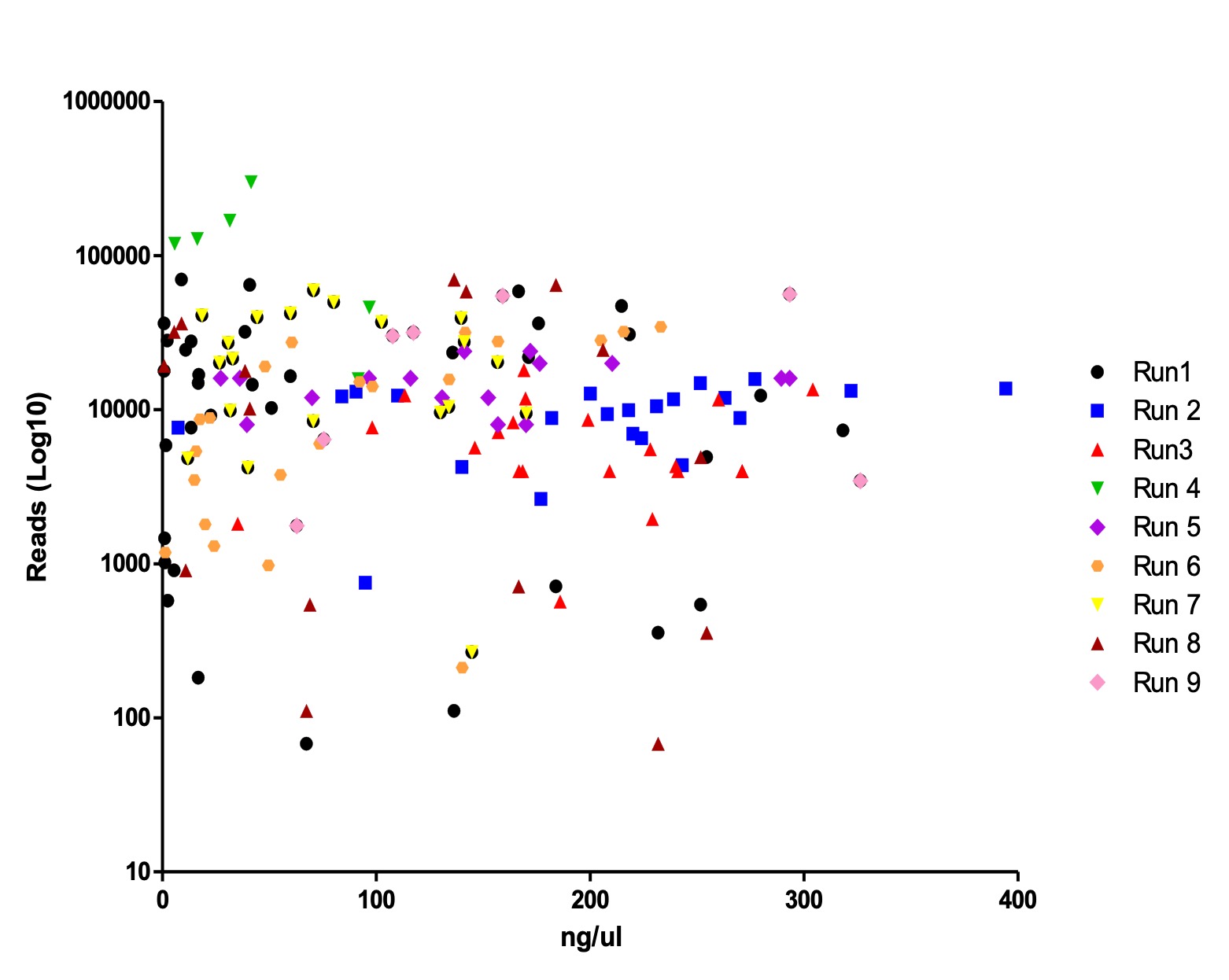

Supplement: Fig. S1 — DNA concentration vs. number of reads. [file jcm.01670-24-s0002.jpg]

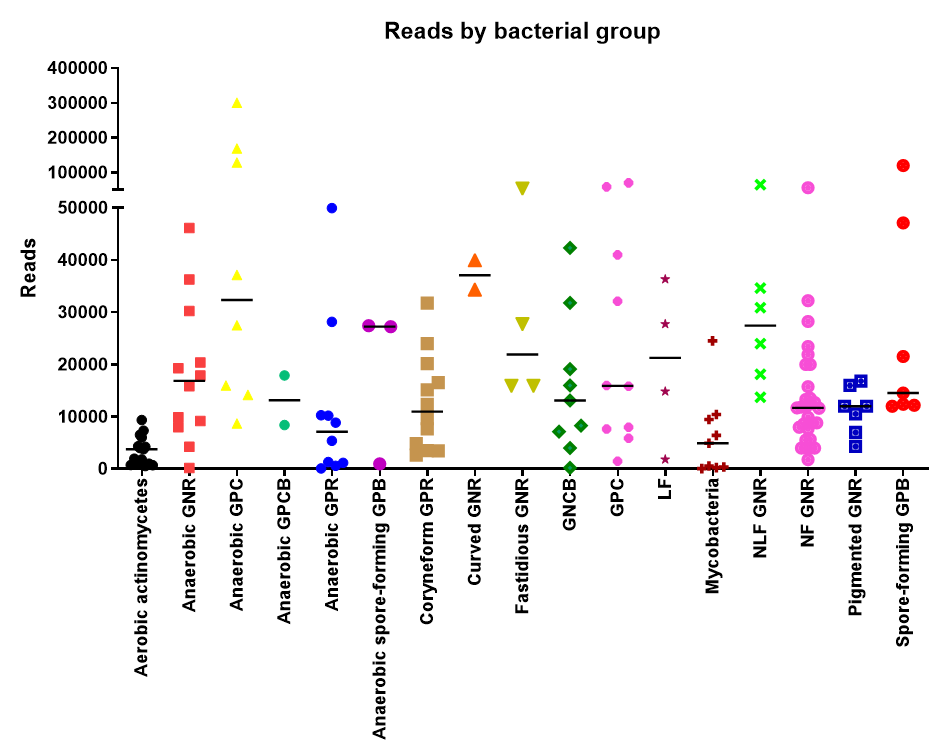

Supplement: Fig. S2 — Number of reads per bacterial group. [file jcm.01670-24-s0003.png]

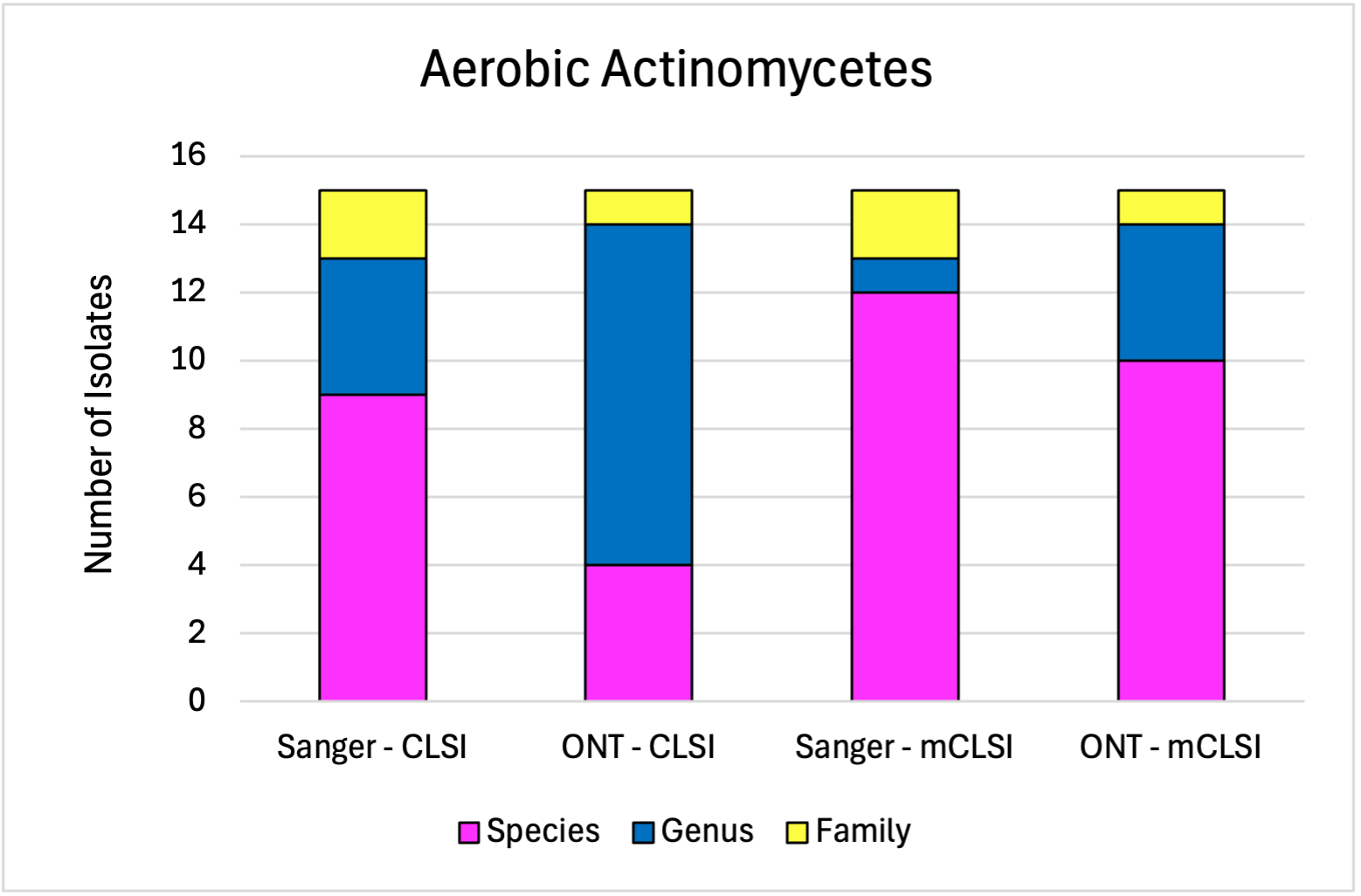

Supplement: Fig. S3 — Level of identification for aerobic actinomycetes. [file jcm.01670-24-s0004.png]

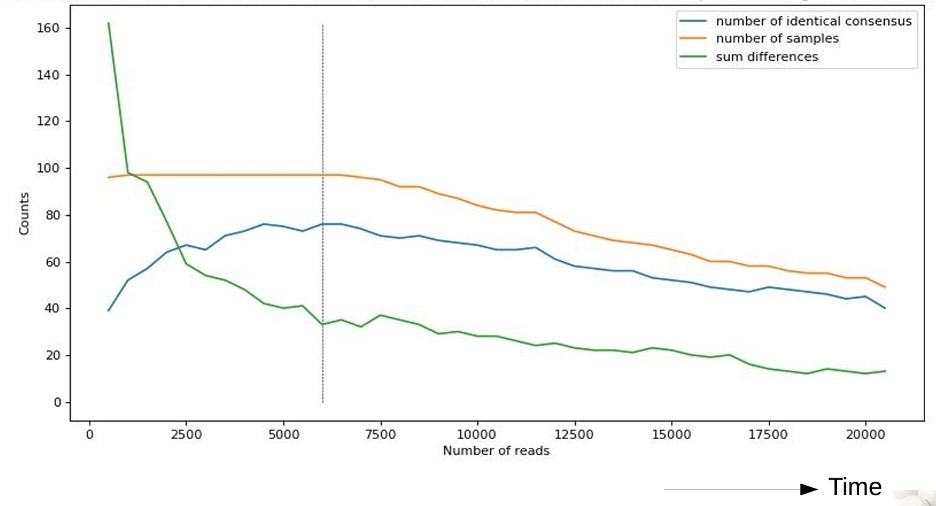

Supplement: Fig. S4 — Differences in consensus based on the number of reads. [file jcm.01670-24-s0005.jpg]
