# Supplementary material for: Evaluation of long-read 16S rRNA next-generation sequencing for identification of bacterial isolates in a clinical diagnostic laboratory
Source: J Clin Microbiol. 2025 Apr 22;63(5):e01670-24. doi: 10.1128/jcm.01670-24 (PMC12077174; doi:10.1128/jcm.01670-24)
Supplement: Supplemental legends — Legends for Fig. S1 to S4. [file jcm.01670-24-s0006.docx]

**Figure S1. Correlation between DNA concentration and number of reads obtained for each sequencing run by 16S ONT sequencing.**

**Figure S2. Number of reads obtained after 16S ONT sequencing by bacterial group**

**Figure S3. Level of identification obtained for aerobic actinomycetes by 16S SS and 16S ONT applying CLSI MM18 A2 (CLSI) and modified-CLSI (mCLSI) guidelines for interpretation.**

**Figure S4. Evolution of the number of identical consensuses, sum of the differences and number of samples according to the number of reads. Blue line: for 67 samples, the consensus achieved with 6000 reads was the same as the consensus obtained with the total number of reads. Seventeen samples had a different consensus (maximum was 4 nucleotide difference) but none of these differences changed the identification.**
